# Piezo1 activation in endothelial cells aggravates microvascular ischemia–reperfusion injury in limbs by enhancing ferroptosis

**DOI:** 10.1038/s12276-025-01616-9

**Published:** 2026-01-09

**Authors:** Fan-feng Chen, Yin-he Zhang, Zi-chang Wu, Kaiyi Du, Xinyuan Chen, Yang Lu, Qianqian Hu, Anyu Du, Shenghu Du, Jian Wang, Keqing Shi, Zimiao Chen, Zili He, Kailiang Zhou, Jian Xiao

**Affiliations:** 1 Department of Vascular Surgery and Department of Wound Healing, The First Affiliated Hospital of Wenzhou Medical University, Wenzhou, China; 2 National Key Clinical Specialty (General Surgery), The First Affiliated Hospital of Wenzhou Medical University, Wenzhou, China; 3 Oujiang Laboratory (Zhejiang Lab for Regenerative Medicine, Vision and Brain Health), School of Pharmaceutical Sciences, Wenzhou Medical University, Wenzhou, China; 4 Department of Orthopaedics, The Second Affiliated Hospital and Yuying Children’s Hospital of Wenzhou Medical University, Wenzhou, China

**Keywords:** Lipid signalling, Peripheral vascular disease, Mechanisms of disease, Metabolomics

## Abstract

Acute limb ischemia–reperfusion injury (ALIRI) prominently involves microvascular dysfunction, with notable contributions from damage to microvascular endothelial cells (MECs). Previous research suggests that the mechanosensitive ion channel Piezo1 becomes active in response to mechanical stress conditions, including ischemia and trauma. However, its precise function within the ALIRI context remains elusive. Notably, the expression of Piezo1 was markedly elevated postreperfusion in mouse hind limb ischemia/reperfusion (I/R) models, implicating its crucial involvement in limb survival. Employing specific inhibitors of cell death pathways, the study delineated key molecular drivers of ferroptosis during limb damage. Here evaluations of limb vitality, western blot, quantitative PCR and immunofluorescence implicated that activation of Piezo1 by its agonist exacerbates I/R-induced microvascular perfusion deficits, tissue swelling, skeletal muscle damage and increased tissue infarction and MECs damage. Conversely, these detrimental impacts were mitigated through pharmacological blockade of Piezo1 or specific deletion of *Piezo1* in MECs. Comprehensive untargeted metabolomic analysis revealed significant changes primarily in glycerophospholipid and arachidonic acid metabolism pathways. Further experiments demonstrated that RNA interference-mediated inhibition of cytosolic phospholipase A2 (cPLA2) and acyl-CoA synthetase long-chain family member 4 (ACSL4) negated the protective effects against ferroptosis and limb damage that were observed with Piezo1 deletion. Moreover, this study confirmed that protein kinase C phosphorylates ACSL4, which mediates Piezo1-induced ferroptosis and exacerbates limb damage, as shown through immunoprecipitation studies. In summary, Piezo1 contributes to the exacerbation of microvascular and skeletal muscle damage in ALIRI by facilitating the cPLA2-dependent release of arachidonic acid and promoting ACSL4-driven lipid peroxidation, thereby intensifying ferroptosis in MECs.

## Piezo1 activation worsens limb damage in ischemia reperfusion

Acute limb ischemia–reperfusion injury (ALIRI) is a serious condition that can occur after blood flow is restored to a limb. This can cause damage to small blood vessels and tissues. Here researchers wanted to understand how a protein called Piezo1 affects this process. The researchers created a model of ALIRI in mice and observed the effects of Piezo1 on cell death and tissue damage. They found that Piezo1 activation increases calcium levels in cells, which then triggers a series of reactions leading to cell death through a process called ferroptosis. They also discovered that inhibiting Piezo1 reduced tissue damage and cell death. The study concludes that targeting Piezo1 could be a potential strategy to prevent tissue damage in ALIRI. Future research may focus on developing treatments that inhibit Piezo1 to improve outcomes for patients with this condition.

This summary was initially drafted using artificial intelligence, then revised and fact-checked by the author.

## Introduction

Acute limb ischemia–reperfusion injury (ALIRI) is a prevalent and grave complication following vascular surgical procedures, characterized by damage to the microvascular systems^1,2^. The rapid restoration of circulation after ischemia can easily cause microvascular blood flow disorders, leading to tissue swelling and increased fascial compartment pressure^3,4^. Without timely intervention and effective treatments, these pathological changes aggravate microcirculatory disturbances and trigger a vicious cycle of ‘ischemia–edema–ischemia’^5^, which can ultimately accelerate the onset of acute compartment syndrome (ACS). Previous investigations have shown that microvascular endothelial cell (MEC) death is a critical factor contributing to the deterioration of ALIRI^6^. Therefore, targeting MEC death during the ischemia/reperfusion (I/R) phase is essential for preventing the progression of ACS following ALIRI^7^.

The core pathogenesis of ALIRI involves a lipid peroxidation cascade triggered by reactive oxygen species (ROS). It has been well-documented that both genetic and pharmacological interventions aimed at modulating the lipid peroxidation cascade can significantly alleviate the damage associated with I/R^8,9^. Notably, the long-chain member of the acyl-CoA synthetase family 4 (ACSL4) catalyzes the binding of polyunsaturated fatty acids (PUFAs) such as arachidonic acid (AA) to CoA, thereby generating PUFA–CoA derivatives^10,11^. These serve as crucial precursors for lipid peroxidation products. As the most abundant, biologically active and extensively distributed PUFA in human tissues, the accumulation of AA is closely associated with lipid peroxidation-mediated cell damage observed in various pathological conditions^12–15^^.^ The activation of ACSL4, which is pivotal for lipid peroxidation, is reportedly regulated by the Ca²⁺-dependent protein kinase C (PKC) signaling pathway^16^. Furthermore, inhibiting the expression and activity of ACSL4 has been shown to alleviate I/R-induced injury^17^. Despite this, the explicit role of AA-mediated lipid peroxidation in contributing to MECs damage within the context of ALIRI is still under investigation and not fully defined.

Mechanotransduction encapsulates the transformation of mechanical stimuli into biochemical signals, a critical biological function that allows organisms to detect and respond to physical influences from their surrounding and internal milieu^18,19^. During episodes of ALIRI, multiple mechanical stresses such as intravascular fluid shear forces, increased hydrostatic pressures and external pressures from swelling tissues, as well as the swelling of MECs themselves, can lead to changes in the tension across MEC membranes. Piezo1 is a nonselective ion channel activated by mechanical force, first identified by Coste and colleagues in 2010^20^. It is highly expressed in various cells and tissues of the cardiovascular, skin and musculoskeletal systems^21–23^. The transmembrane ion channel protein is activated by mechanical stress^19^, which opens the channel allowing Ca^2+^ to enter and activate calpain signaling pathways that are dependent on Ca^2+^ (ref. ^24^). The activation of the Piezo1 channel has been identified as a consequence of mechanical stress changes caused by shifts in cellular ion homeostasis and an increase in cell membrane surface area^25^. The opening of this channel importantly impacts various physiological and pathological conditions, including mechanical stimulation of MECs, nitric oxide (NO) synthesis, lipid metabolism, DNA damage prevention and promotion of cell motility^26^. Nevertheless, the precise role of Piezo1 together with the involvement of Ca²⁺ signaling in mediating MEC damage during ALIRI remains unclear and is currently under investigation.

To this end, the involvement of the mechanosensitive ion channel Piezo1 in the microvascular and skeletal muscle tissues injury after ALIRI was investigated in this work using a mouse model with tissue-specific deletion of Piezo1. Piezo1 activation in MECs was shown to lead to a series of molecular events, the first of which is an increase in Ca²⁺ ions inflow. This influx is important for triggering the release of AA via activation of the Ca^2+^-dependent cytosolic phospholipase A2 (cPLA2) pathway. At the same time, it enhances the lipid peroxidation processes connected with AA through the Ca²⁺-dependent PKC–ACSL4 pathway. This work not only provided evidence for the mechanistic pathways in vivo, but also supported these observations by performing corresponding in vitro assays. The systematic approach has therefore elucidated the pathways that are involved in Piezo1-mediated MECs ferroptosis during ALIRI and has identified potential therapeutic target.

## Materials and methods

### Animals and ethics statement

The entire experimental methods and procedures followed the Animal Care and Use Committees guidelines approved by the Animal Research Ethics Committee of Wenzhou Medical University (wydw 2023-0248). Male endothelial-specific deletion of *Piezo1* (*Piezo1*^*EKO*^) and C57BL/6 mice were obtained from Beijing Vital River Laboratory Animal Technology Co. Ltd. These animals were raised in the specific pathogen-free conditions, with free access to food and water and the ability to move around freely according to the guidelines for animal experimentation.

### Model of ALIRI

For the induction of ALIRI, an orthodontic rubber band was tightly secured around the greater trochanter of the right leg, maintaining this setup for 4 h to create a state of ischemia. This was followed by a 24-h period of reperfusion to simulate recovery. An identical process was applied to the mice in the sham group, excluding the application of the orthodontic rubber band. After reperfusion, all mice were subjected to laser Doppler imaging (LDI) under general anesthesia.

### Groups, establishment and treatments

*Piezo1*^*EKO*^ mice were allocated into the sham group, I/R group, I/R + erastin group, I/R + adeno-associated virus (AAV)-scramble group, I/R + AAV-cPLA2 group, I/R + AAV-ACSL4 group and I/R + AAV-PKCβII group. C57BL/6 mice were distributed into sham group, I/R group, I/R + 3-methyladenine (3-MA) group, I/R + necrostatin-1 (Nec-1) group, I/R + Z-DEVD-FMK group, I/R + ferrostatin-1 (Fer-1) group, I/R + Yoda1 group, I/R + GsMTx4 group, I/R + erastin group, I/R + AAV-Scramble group, I/R + AAV-cPLA2 group, I/R + AAV-ACSL4 group and I/R + AAV-PKCβII group. Mice were intraperitoneally injected with various inhibitors of cell death the day before I/R surgery and subsequently every day after surgery: 3-MA (15 mg/kg), Nec-1 (1 mg/kg), Z-DEVD-FMK (1.8 mg/kg) and Fer-1 (1 mg/kg). In the GsMTx4 or Yoda1 group, the mice were intraperitoneally injected with 1 mg/kg GsMTx4 or 5 μmol/kg Yoda1, respectively, every other day starting on the day of the ALIRI surgery. Mice from I/R + erastin group were treated with the intraperitoneal injections of erastin (10 mg/kg) once a day with the protocol. For the I/R + AAV-cPLA2, I/R + AAV-ACSL4 and I/R + AAV-PKCβII groups, direct intramuscular injections of AAV-cPLA2, AAV-ACSL4 or AAV-PKCβII, respectively, were administered at three different locations in the gastrocnemius on the right 2 weeks before the operation. An equivalent number of AAV vectors carrying a negative control sequence was administered to the control group.

### LDI

After receiving anesthesia, mice designated to the sham or experimental model groups were promptly transferred to an environment that adhered to standard research protocols. The perfusion metrics of the hind limbs were meticulously recorded using a LDI system, specifically the Moor LDI 5061 model, operated with Moor Software Version 3.01 (Moor Instruments). For accuracy and reliability, each mouse was assessed three separate times, and the mean of these recordings was calculated to serve as the basis for subsequent statistical evaluations.

### Determination of muscle tissue edema

Upon retrieval of muscle tissues from the experimental mice, these specimens were immediately subjected to a blotting process for initial moisture removal and then accurately weighed. Subsequently, these tissues were placed within a controlled heating environment, specifically an oven set to maintain a temperature of 65 °C. The tissues remained in this environment until they achieved a consistent dry mass. The assessment of muscle tissue edema was conducted by establishing the ratio of the initial wet weight to the final dry weight, providing a quantitative measure of fluid retention.

### RNA interference

Within our animal experiments, the following specific AAVs, which specifically target MECs, were developed: AAV-cPLA2, AAV-ACSL4 and AAV-PKCβII; these were produced and packaged by GeneChem Chemical Technology Co. Ltd. AAV-ACSL4 Thr328Ala were produced and packaged by Bioscien. Concurrently, we generated rAAVs intended for the delivery of three murine genes cPLA2, ACSL4 and PKCβII. These genes were placed under the control of an endothelium-specific promoter (ICAM-2) to ensure targeted expression. For local administration, these AAVs were injected intramuscularly into the gastrocnemius muscle at the hind limb, delivering a dose of 5 × 10¹⁰ viral genomes per injection site. This administration was performed 14 days before any surgical intervention to allow adequate time for viral integration and gene expression.

GenePharma synthesized specific short interfering RNAs (siRNAs) used to target Piezo1 in the transfection of human umbilical vein endothelial cells (HUVECs). For the transfection procedure, the liposome-based delivery system, Liposome Reagent 3000, was employed to facilitate the efficient incorporation of these siRNAs into the cellular framework. At 48 h post-transfection, the success of *Piezo1* silencing was rigorously evaluated through qPCR to verify the reduction in gene expression levels.

### Cell culture

HUVECs sourced from Wenzhou Medical University were propagated in Dulbecco’s modified Eagle medium supplemented with essential nutrients, routinely passaged upon reaching 80–90% confluency. For the oxygen–glucose deprivation/reoxygenation (OGD/R) studies, the cells were utilized during their logarithmic growth phase. Initially, the culture medium was exchanged with a solution devoid of fetal bovine serum and glucose to induce hypoxia. Subsequently, the culture vessels were placed inside a sterilized hypoxic chamber maintained at 37 °C. The atmosphere within the chamber was controlled to contain 1% oxygen, 95% nitrogen and 5% carbon dioxide for 4 h. Cells pretreated with pharmacological agents such as Yoda1 (5 μM), GsMTx4 (2.5 μM), AA (20 μM) or EGTA-AM (20 mM) were then subjected to OGD/R. Post-treatment cell viability was quantitatively assessed using the CCK-8 assay, with results expressed as OD at 450 nm ratios.

### Antibodies and reagents

In the course of our research, we employed a diverse array of chemicals and reagents from several reputable sources. Specifically, inhibitors of cell death were obtained from Selleck Chemicals, which included Fer-1 (S7243), Nec-1 (S8037), Z-DEVD-FMK (S7312) and 3-MA (S2767). Additional compounds such as Yoda1 (HY-18723), GsMTx4 (HY-P1410), erastin (HY-15763) and EGTA-AM (HY-D0973) were acquired from MedChemExpress. From Shanghai Aladdin Biochemical Technology Co. Ltd., we sourced AA (A111764). Our histological studies utilized Masson Staining Kit (G1340) and the 2,3,5-triphenyltetrazolium chloride (TTC) Staining Kit (G3005) obtained from Solarbio Science & Technology. From Abcam, we procured the 4-HNE Assay Kit (ab238538) and the lipid hydroperoxide (LPO) Assay Kit (ab133085). Beyotime Biotechnology supplied us with kits for assessing malondialdehyde (MDA, S0131S), ROS (S0033S), JC-1 (C2006) and protein quantitation via the bicinchoninic acid (P0010S) method. An AA ELISA Kit (CB10353-Mu) was sourced from Shanghai Enzyme-linked Biotechnology Co. Ltd. For immunodetection, specific primary antibodies such as GAPDH (10494-1-AP) were purchased from Proteintech Group. Antibodies targeting Piezo1 (DF12083), cPLA2 (AF6329) and its phosphorylated form (AF3329) were obtained from Affinity Biosciences. Santa Cruz Biotechnology provided antibodies for ACSL4 (sc-365230), PKCβII (sc-13149), phosphorylated PKCβII (sc-365463), vascular endothelial cadherin (VE-cadherin or CDH5, sc-9989) and platelet endothelial cell adhesion molecule-1 (CD31, sc-376764). Receptor-interacting serine/threonine-protein kinase 1 (RIPK1, R40018), receptor-interacting serine/threonine-protein kinase 3 (RIPK3, R30283), mixed lineage kinase domain-like protein (MLKL, R380559) and glutathione peroxidase 4 (GPX4, 381958) were obtained from ZENBIO. Alpha-smooth muscle actin (α-SMA, GB111364-100) and cluster of differentiation 68 (CD68, GB113109-100) were obtained from Servicebio. Abcam also supplied the phosphorylated serine/threonine antibody (P-Ser/Thr, ab-17464), and 4-HNE (MAB3249) was from R&D Systems. The horseradish peroxidase (HRP)-conjugated secondary antibodies were sourced from Santa Cruz Biotechnology, while the 4′,6-diamidino-2-phenylindole (DAPI) solution was obtained from Beyotime Biotechnology.

### RNA-sequencing library preparation, sequencing and analysis

LC-Biotechnologies undertook the task of extracting total RNA, which was designated for comprehensive transcriptomic studies. After extraction, the differentially expressed mRNAs were identified using advanced bioinformatic algorithms in the edgeR and DESeq2 software packages. These tools identified mRNAs that demonstrated a significant fold change (FC), specifically those with FC over 2 or below 0.5. Statistical significance was rigorously determined by a threshold of *P* value less than 0.05, ensuring that only mRNAs with substantial variations in expression levels were highlighted for further investigation.

### Real-time quantitative PCR

Total RNA was extracted using Trizol, and cDNA was synthesized with TransScript One-Step SuperMix, combining genomic DNA removal and cDNA synthesis in a single step. This cDNA was subsequently employed as the template for reverse transcription reactions. Real-time PCR was executed utilizing specific primers and was performed on a nexus GSX1 Mastercycler (Eppendorf). The PCR reaction was carried out in a final volume of 20 μl. The thermal cycling conditions included an initial incubation at 50 °C for 120 s to ensure optimal enzyme activity, followed by a denaturation step at 95 °C for 20 s. Forty PCR cycles were performed, and data were analyzed using the ${2}^{-\mathrm{\varDelta \varDelta }{C}_{t}}$ method, normalizing target gene expression to β-actin. The following gene-specific primers utilized for real-time PCR were synthesized and provided by GENERAY Biotechnology: *Piezo1*, 5′-ATCGCCATCATCTGGTTCCC-3′ (For) and 5′-TCTGTGCAGTGACGATGTCC-3′ (Rev); *Acsl4*, 5′-ACTGGCGATATTGGAGAAT-3′ (For) and 5′-CACATAGGACTGGTCACTT′ (Rev); *Gpx4*, 5′-CAGGAGCCAGGAAGTAAT-3′ (For) and 5′-CAGCCGTTCTTATCAATGAG-3′ (Rev).

### Metabolomics data processing and analysis

For the metabolite analysis, all samples were acquired by liquid chromatography–mass spectrometry following the machine instructions. An ACQUITY UPLC T3 column (Waters) was used for the reversed-phase separation. Chromatographic separation conditions were as follows: column temperature 40 °C and flow rate 0.3 ml/min. Using an UltiMate 3000 UPLC System (Thermo Fisher Scientific), metabolites eluted from the column were detected. Gradient elution conditions were set as follows: 0–0.8 min, 2% B; 0.8–2.8 min, 2% to 70% B; 2.8–5.6 min, 70% to 90% B; 5.6–6.4 min, 90% to 100% B; 6.4–8.0 min, 100% B; 8.0–8.1 min, 100% to 2% B; 8.1–10 min, 2% B.

A high-resolution tandem mass spectrometer Q-Exactive (Thermo Scientific) was used to detect metabolites eluted from the column. The Q-Exactive was operated in both positive and negative ion modes. Precursor spectra (70–1,050 *m*/*z*) were collected at a resolution of 7 × 10^4^ resolution with an AGC target of 3 × 10^6^. The maximum inject time was set to 100 ms. A top three configuration to acquire data was set in DDA mode. Fragment spectra were collected at 1.75 × 10^4^ resolution to hit an AGC target of 1 × 10^5^ with a maximum inject time of 80 ms. To evaluate the stability of the liquid chromatography–mass spectrometry during the whole acquisition, a quality control sample (a pool of all samples) was acquired after every ten samples.

For data processing and analysis, raw data files were converted into mzXML format and then processed by XCMS, CAMERA and metaX, and the intensity of peak data was further preprocessed by metaX. A principal component analysis was performed for outlier detection and batch effects evaluation using the preprocessed dataset. Supervised partial least squares discriminant analysis (PLS-DA) was conducted through metaX to discriminate the different variables between groups. The online Kyoto Encyclopedia of Genes and Genomes (KEGG) database was used to annotate the metabolites by matching the exact molecular mass data (*m*/*z*) of samples with those from the database. To screen significantly different metabolic markers, univariate statistical analysis with the criteria of variable importance in the projection >1 and FC of metabolites less than 0.5 or more than 2, coupled with a *P* value <0.05 was used, which was visualized by volcano plots and heat maps^27,28^. The KEGG database was used to determine the relevant significant changed metabolism pathway.

### Immunofluorescence staining of HUVECs and gastrocnemius muscle slices

HUVECs were fixed with 4% paraformaldehyde, permeabilized with Triton X-100 and then incubated with antibodies against 4-HNE and dihydroethidium (DHE). For the detection of markers that are associated with oxidative stress, Alexa Fluor conjugated secondary antibodies were used. To label and visualize the cell nuclei, the cells were stained with DAPI. This staining protocol facilitates the identification of oxidative stress markers and morphology of the cells under fluorescence microscopy.

The parts of the gastrocnemius muscle were stained to identify certain markers. The staining process was done with the help of primary antibodies specific to Piezo1, CD31, α-SMA, CD68, CDH5, p-cPLA2 and ACSL4 to label these proteins in the sections. The cases after the primary antibody incubation were treated with secondary antibodies conjugated to Alexa Fluor dyes to visualize the primary antibody binding. DAPI was used to stain the specimens to facilitate nuclear visualization. The sections were then further analyzed under a Nikon ECLIPSE 80i microscope to obtain high-resolution images of the fluorescent markers and other cellular elements.

### Cell culture in polyacrylamide gels

To prepare the initiator stock solution, 0.05 g of lithium phenyl (2,4,6-trimethylbenzoyl) phosphinate was dissolved in 20 ml of phosphate buffered saline (PBS) in a dark brown bottle and incubated in a 50 °C water bath for 15 min. According to the manufacturer’s protocol, gelatin methacryloyl (GelMA) and the initiator stock solution were mixed in a centrifuge tube, vortexed and then dissolved in a 70 °C water bath under dark conditions for 30 min to obtain the GelMA solution. Cultured cells were digested, centrifuged and the resulting cell pellet was resuspended in the preheated GelMA solution to form a cell suspension. The suspension was quickly transferred into a 12-well plate and polymerized using a curing light for about 20 s to solidify the gel. Culture medium was then added and the gels were incubated at 37 °C in a 5% CO₂ atmosphere for 24 h. For gel lysis, a GelMA lysis buffer working solution (0.3 mg/ml) was added to the wells and incubated at 37 °C for 2 h. Lysis was performed at 37 °C for 2 h. The samples were centrifuged at 1,000 rpm for 5 min and the supernatant was discarded. Cell lysis buffer was subsequently added to extract proteins for western blot analysis.

### Immunoprecipitation

To conduct the western blot analysis, the cells were first lysed using an immunoprecipitation buffer that would solubilize the proteins of interest but at the same time maintain their interactions. After that, the cell lysates were mixed with specific antibodies and protein A/G beads. This was followed by overnight incubation to ensure that the antibodies bound to the target proteins, after which the complexes were captured using the protein A/G beads. After this immunoprecipitation, the samples were washed to remove any other proteins that may have been bound to the beads nonspecifically and other contaminants. The last stage was to boil the samples in SDS buffer, which leads to the denaturation of proteins and degradation of the complexes to enable the samples to be analyzed by western blot.

### Western blotting

First, the tissues or cells were lysed to obtain proteins in solution from the muscle tissue samples. The amount of protein in the samples was then estimated to determine the concentration of protein extracted from the samples. SDS–PAGE was then used to separate the proteins. After electrophoresis, the proteins were transferred onto a polyvinylidene difluoride membrane for further analysis. The membranes were then probed with antibodies that specifically recognize the proteins of interest to enable their detection. The protein bands on the membrane were further analyzed with Image Lab software for band intensity and protein expression level quantification, which allows for a deeper interpretation of the results.

### Ca^2+^ imaging

HUVECs were then seeded onto coverslips and then exposed to fluo-4 acetoxymethyl ester (Fluo-4 AM, G1724-100T, Servicebio), a Ca^2+^ sensitive dye that helps in tracking changes in intracellular Ca^2+^ levels. The cells were then stained with Fluo-4 AM and then the cells were observed under a confocal microscope to detect the fluorescence intensity. The confocal microscope was set to excite the dye at 340 nm and 380 nm, with a 5-s interval between acquisitions. This arrangement enabled the continuous analysis of kinetics in the fluorescence intensity change. Imaging was done using AxioVision software, in which the fluorescence signals were captured and analyzed. To eliminate the background noise, the readings were corrected by subtracting the background fluorescence. The final results appear as the ratio of the fluorescence intensities at 340 nm to that of 380 nm and these were presented as mean ± s.d. for each of the groups.

### AFM

Following anesthesia, gastrocnemius muscle tissue was collected and gently blotted with absorbent paper to remove surface moisture. The tissue was embedded in OCT and sectioned at 20 µm thickness using a cryostat (Cryostar NX70, Thermo Fisher). Sections were mounted on glass slides for atomic force microscopy (AFM) analysis. AFM measurements were performed using a commercial AFM system (NanoWizard, Bruker Nano Inc.) in force-mapping mode. Both topographical and Young’s modulus images were acquired to characterize the structural and mechanical properties of the samples. To maintain tissue hydration, a drop of enzyme-free PBS was applied to each section before measurement. For Young’s modulus distribution maps of the sham and I/R group samples, scans were performed over a 20 µm × 20 µm area, with 64 × 64 force-indentation curves recorded.

### Detection of AA in mice

To prevent the mice from moving or causing themselves any discomfort during the process, the mice were anesthetized and then received a perfusion with a saline solution to wash out any remaining blood in their bodies. This is important to avoid any kind of interaction from the blood components in the following experiments. Gastrocnemius muscles were then removed from the animals in a manner that was least invasive to the body. Muscle tissues were homogenized to obtain muscle lysates to ensure that the muscle matrix was fully disrupted to release contents within the cells. These lysates were used for determining the free AA levels using ELISA that was developed for mouse samples. The ELISA procedure involved several critical steps. First, the samples were incubated with antibodies labeled with HRP and these antibodies selectively interact with AA. This was then followed by the addition of chromogen solution, which reacts with HRP to give a color which is proportional to the concentration of AA. To cease the enzymatic activity and to fix the color, a stop solution was then added. Finally, the resulting color intensity was quantitatively assessed by measuring the absorbance at a wavelength of 450 nm by using a spectrophotometric plate reader. This measurement enabled the accurate determination of free AA concentrations in the muscle samples, which were then used for other analysis.

### Masson staining

A histological examination was performed on the gastrocnemius muscles. First, the muscle tissues were fixed to maintain the structural integrity of the cells. The tissues were then embedded in paraffin wax so they could be sectioned easily. The muscle samples were then fixed and embedded in paraffin and thin sections of the muscle were sliced and put on slides. For this purpose, these sections were stained with Masson trichrome staining to see the muscular tissues and check for any structural changes. This staining technique helps in the identification of muscle fibers and connective tissue. Thus, an assessment of muscle fiber damage was performed. The extent of damage was determined and expressed as a percentage of the total muscle fibers cross-sectional area. Muscle damage was evaluated using a scoring system based on the percentage of injured fibers in the tissue sections.

### TTC staining

After allowing a 24-h reperfusion period in the mice, the gastrocnemius muscles were carefully excised. The initial step involved washing the muscle tissue surfaces thoroughly with ice-cold PBS to remove any residual blood or debris. Subsequently, the tissues were rapidly frozen at −80 °C for 3 min to preserve their structure for further processing. Once frozen, the muscles were transversely sectioned into slices 1–2 mm thick, from the ligation site to the Achilles tendon, to ensure uniformity. The muscle sections were then subjected to staining with 1% TTC solution, incubated at 37 °C for 15–20 min. TTC staining is a crucial technique for differentiating between infarcted and viable tissues; the infarcted regions, which have reduced enzymatic activity, do not take up the stain and thus appear white, while the normal, viable muscle tissue turns red. Following staining, the sections were fixed in a 4% (w/v) paraformaldehyde solution for a period of 24 h to preserve the staining pattern and tissue morphology. To assess the extent of muscle damage, the infarction volume was measured by calculating the proportion of the unstained (white) area relative to the total muscle tissue area in each section. This measurement provided a quantitative evaluation of the infarcted region. The results were expressed as a percentage of the infarcted volume relative to the total volume of muscle tissue, allowing for a detailed assessment of the muscle damage.

### Measurement of ROS level

Skeletal muscle cryosections were used in two different assays to determine the intracellular ROS levels. First, the ROS level was investigated using a ROS assay kit to determine the quantify the ROS present in the cells. Second, the sections were stained with DHE, a fluorescent dye that reacts with superoxide radicals and produces fluorescence, hence indicating ROS production. Last, the samples were analyzed by confocal microscopy. This imaging technique offers clear images of the stained muscle tissues, allowing the analysis of the distribution and density of ROS in the cells. The two methods, which include the ROS assay kit and DHE staining, enable the assessment of the level of oxidative stress on the skeletal muscle tissues effectively.

### LPO detection

Concentrations of 4-HNE were measured in both cells and muscle tissue using the 4-HNE assay kit from Abcam with adherence to the manufacturer’s guidelines. For the detection of MDA levels in cells and muscle tissues, the MDA assay kit from Beyotime was used. Further, the LPO was also assessed using the LPO assay kit from Abcam.

### JC-1 staining

To evaluate ferroptosis within cells, JC-1 dye was employed, which allows for the differentiation of mitochondrial JC-1 monomers emitting green fluorescence from JC-1 aggregates emitting red fluorescence. This differential fluorescence was observed and recorded using fluorescence microscopy. Subsequent analysis involved quantifying the red/green fluorescence ratios using ImageJ software to assess mitochondrial membrane potential changes.

### Statistical analysis

Statistical evaluations were conducted using SPSS, presenting data as mean ± s.d. Both ANOVA and subsequent post hoc tests, along with *t*-tests, were utilized to assess statistical significance, with a threshold of *P* < 0.05 for determining significance.

## Results

### Piezo1 is upregulated after ALIRI and ferroptosis is the predominant form of cell death in ALIRI

**Fig. 1:**
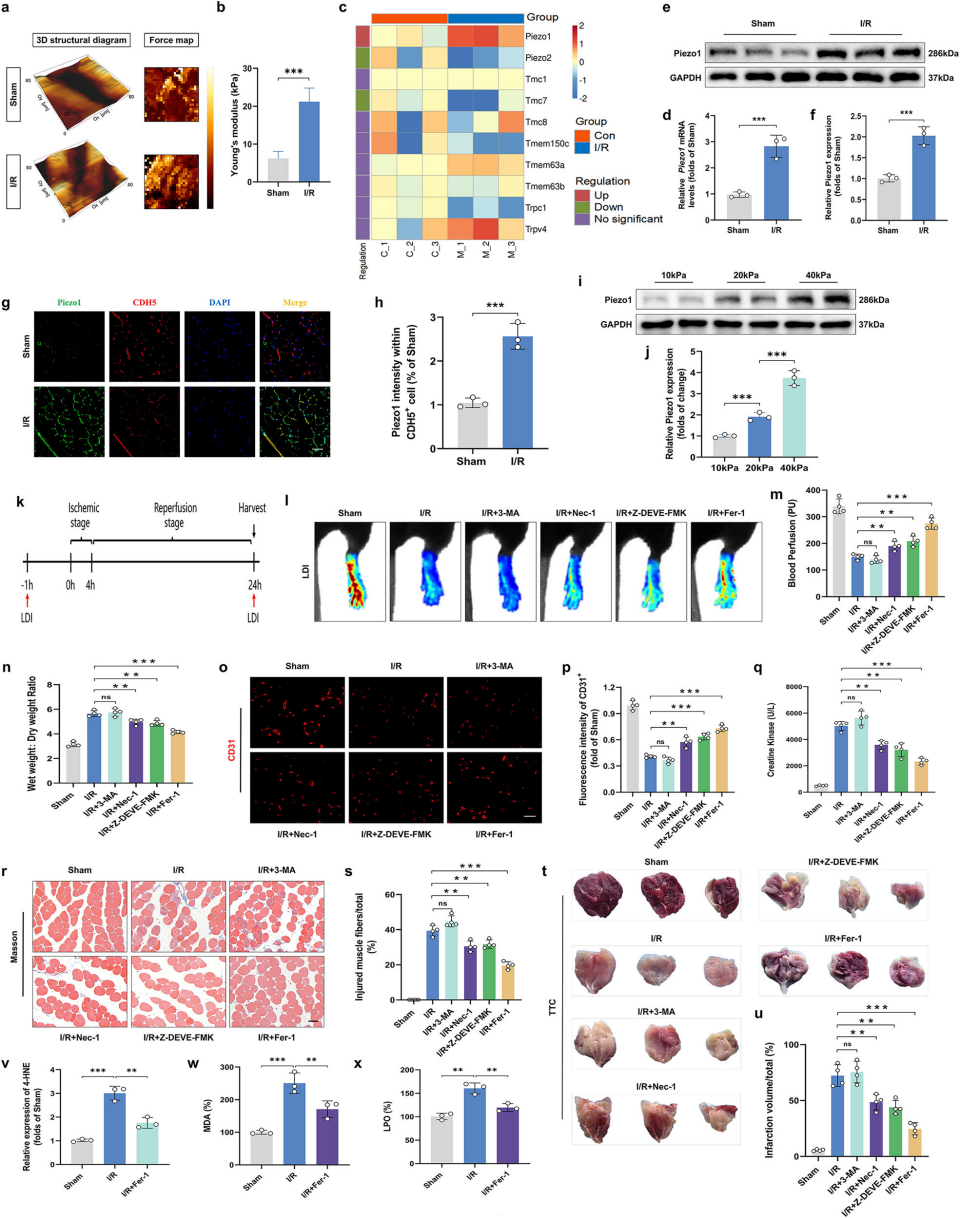
Regulation and impact of Piezo1 in ALIRI with a focus on ferroptosis as the predominant cell death mode. **a**, **b** Representative images showing the 3D structural diagram and force map (**a**) and the statistical analysis (**b**) of the Young’s modulus measured in skeletal muscle tissues. **c** A heat map of expression levels of various ion channels. **d** Analysis of *Piezo1* relative expression. **e** Western blot images showing Piezo1 protein levels. **f** Piezo1 protein quantification relative to GAPDH from the samples shown in **e**. **g** Histological sections of skeletal muscle from mice, immunostaining for Piezo1 and CDH5, with DAPI highlighting nuclei. Scale bars, 100 µm. **h** Dual-positive cell counts for Piezo1 and CDH5 and proportion out of total CDH5-positive cells are quantified. **i** Representative immunoblots of Piezo1 expression in HUVECs seeded on 10, 20 and 40 kPa polyacrylamide gels. **j** Piezo1 protein quantification relative to GAPDH. **k** A schematic representation of the mouse model used for I/R injury. **l** LDI data showing hind limb blood flow post-I/R. **m** A histogram representation of blood flow signal intensity. **n** Measurement of tissue edema through the wet-to-dry weight ratio. **o** Confocal microscopy images of skeletal muscle cross-sections stained for CD31. Scale bars, 100 µm. **p** Immunofluorescence data for CD31 with mean optical density values calculated from the images in **o**. **q** Serum CK levels, indicative of muscle cell death. **r** Masson’s trichrome staining of skeletal muscle cross-sections. Scale bars, 100 µm. **s** Calculation of the proportion of damaged fibers in skeletal muscle samples. **t** TTC staining of skeletal muscle cross-sections to visualize infarction. **u** Overall quantification of infarct volume in the gastrocnemius. **v** Histogram showing the levels of 4-HNE. **w** MDA content shown as a histogram. **x** LPO content shown as a histogram. Values are shown as means ± s.d. ns, no significant difference; statistical significance levels: ^*^P < 0.05, ^**^P < 0.01 and ^***^P < 0.001.

Mechanosensitive ion channels have been implicated in the regulation of cell death, and their disruption is closely linked to various vascular diseases^29,30^. In this study, we focused on analyzing these channels in skeletal muscle tissues subjected to ALIRI. First, AFM was employed to quantitatively assess the Young’s modulus of gastrocnemius muscle tissues from both the sham and I/R groups. The results revealed that the mechanical stress of the gastrocnemius muscle markedly increased after I/R (Fig. 1a, b). As illustrated in the heat map in Fig. 1c, a substantial alteration in the mechanosensitive ion channels was observed following ALIRI. Notably, Piezo1 levels exhibited a remarkable increase among these ion channels. Our investigation further confirmed that this elevated Piezo1 expression is associated with the damage observed in tissues after ALIRI. Specifically, we measured increased mRNA and protein levels of Piezo1 in the mouse gastrocnemius muscle post-ALIRI (Fig. 1d–f). Furthermore, compared with the sham group, the fluorescence intensity of Piezo1 was significantly higher mainly in MECs rather than in fibroblasts or macrophages within skeletal muscle tissues (Fig. 1g, h and Supplementary Fig. 1a, b). To investigate whether the upregulation of Piezo1 following ALIRI is due to stiffness-mediated activation, a variety of fibronectin-conjugated polyacrylamide substrates (10, 20 and 40 kPa) were used to mimic the biomechanical stages during ALIRI. The results demonstrated that matrix stiffness markedly enhanced Piezo1 expression (Fig. 1i, j). Meanwhile, the Fluo-4 AM staining indicated that OGD/R promoted Ca^2+^ influx, which was significantly attenuated by GsMTx4, a highly selective Piezo1 inhibitor (Supplementary Fig. 1c, d). Collectively, these findings demonstrate that Piezo1 is upregulated and activated in ECs and plays a vital role after ALIRI.

Accumulating evidence has shown that I/R-induced tissue and cellular injury involves multiple forms of regulated cell death, including autophagy, apoptosis, necroptosis, ferroptosis, pyroptosis and so on^31^. Our previous studies further confirmed that autophagy, apoptosis and ferroptosis contribute to ALIRI progression^6,32^. Here, we provide additional evidence showing that the necroptosis levels also increased following ALIRI (Supplementary Fig. 1e–h). On the basis of these observations, we designed a series of experiments to comprehensively assess the impact of different forms of cell death on I/R-induced tissue damage in mice (Fig. 1k). Notably, after I/R injury, the group treated with Fer-1 demonstrated superior improvement in limb perfusion compared to all other treatment groups (Fig. 1l, m). Furthermore, an analysis of microvascular density, determined by positive CD31 staining, revealed a significant increase in the Fer-1-treated group (Fig. 1o, p). In addition to vascular protection, Fer-1 treatment was associated with marked improvements in skeletal muscle condition. This was evidenced by a reduced wet-to-dry weight ratio (Fig. 1n) and a lower serum level of creatine kinase (CK), which is a biomarker of muscle damage (Fig. 1q). Masson staining further illustrated that while Nec-1 and Z-DEVE-FMK treatments led to reductions in the proportion of injured muscle fibers, the extent of protection was most pronounced in the Fer-1-treated group, which exhibited a more substantial decrease in fiber injury (Fig. 1r, s). Similarly, TTC staining results indicated that Fer-1 treatment effectively diminished the volume of infarcted regions more than the other treatments (Fig. 1t, u). Finally, our analysis of lipid peroxidation products, such as 4-HNE, MDA and LPO, key markers of ferroptosis, showed that these levels were significantly lower in the Fer-1-treated group (Fig. 1v–x). Taken together, these findings robustly demonstrate that Fer-1 provides substantial and specific protection against both microvascular and skeletal muscle damage induced by ALIRI. To verify whether the upregulation of Piezo1 after ALIRI is associated with ferroptosis, we assessed ferroptosis levels under different stiffness conditions of the matrix gel. The results revealed that ferroptosis levels increased in parallel with rising substrate stiffness (Supplementary Fig. 1i–k). Furthermore, the application of Fer-1 effectively suppressed the ferroptosis exacerbated by Yoda1 (a highly selective agonist of Piezo1) (Supplementary Fig. 1l–n). These findings highlight ferroptosis as the predominant form of cell death in skeletal muscle injury following ALIRI, and underscore the therapeutic potential of targeting the Piezo1-mediated ferroptosis pathway.

### Targeted Piezo1 inhibition modifies ALIRI-induced ferroptosis and microvascular damage

**Fig. 2:**
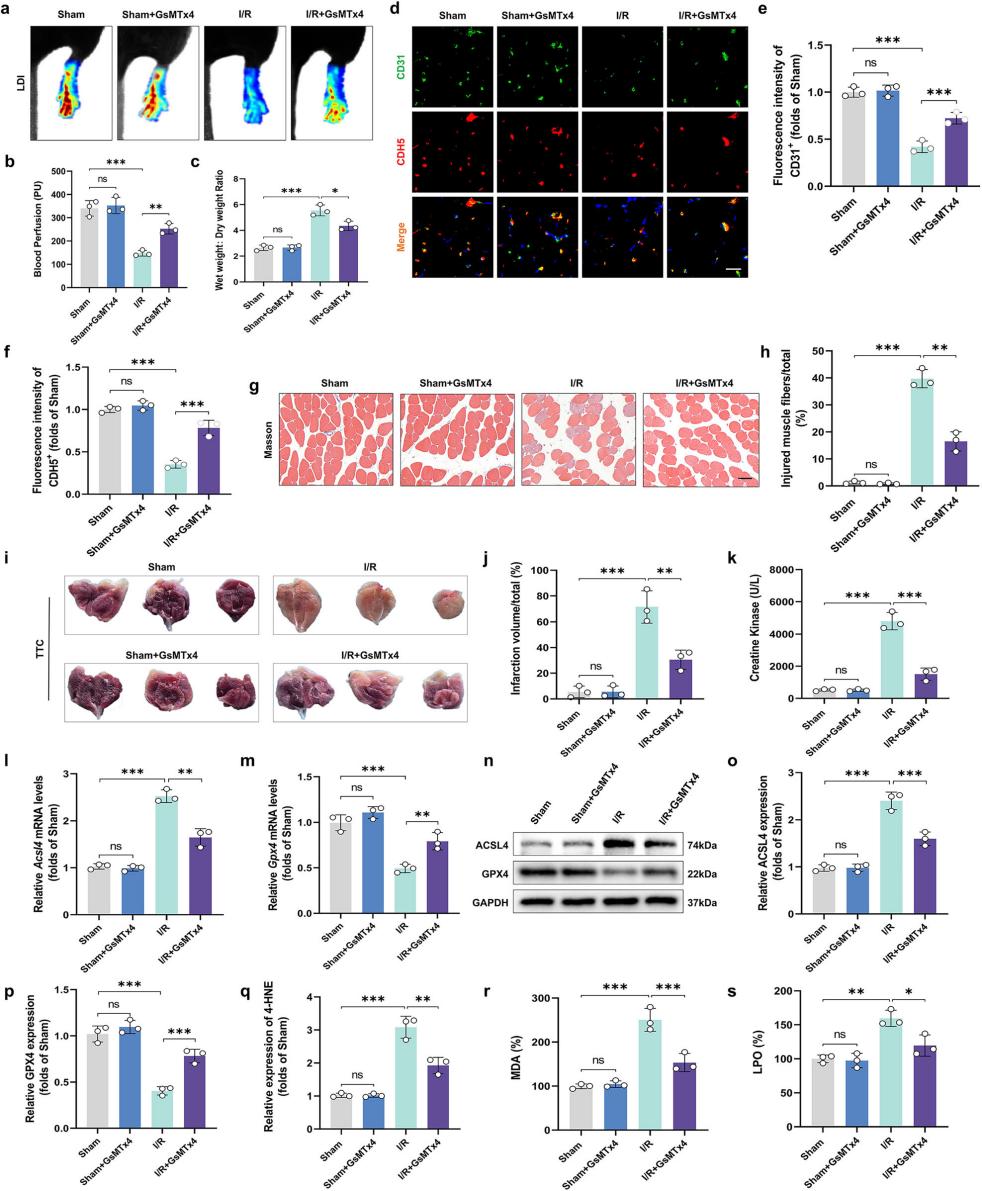
Effects of specific inhibition of Piezo1 on ALIRI-related ferroptosis and microvascular alterations. **a** Blood perfusion in the hind limbs. **b** Histogram depicting the intensity of blood flow signals. **c** Measurement of tissue edema through the wet-to-dry weight ratio. **d** Histological sections of skeletal muscle from mice, immunostaining for CD31 and CDH5. Scale bars, 100 µm. **e**, **f** Quantification of mean optical density values of CD31 (**e**) and CDH5 (**f**). **g** Masson’s trichrome staining of skeletal muscle sections to highlight muscle fiber architecture. Scale bars, 100 µm. **h** Evaluation of the proportion of damaged fibers in skeletal muscle. **i** TTC staining employed to assess the extent of muscle damage in skeletal sections. **j** The total volume of infarction in the gastrocnemius quantified. **k** Serum CK levels. **l**, **m** Analysis of *Acsl4* (**l**) and *Gpx4* (**m**) relative expression. **n** Western blot images showing ACSL4 and GPX4 protein levels. **o**, **p** Quantitative analysis of ACSL4 (**o**) and GPX4 (**p**) proteins, normalized against GAPDH. **q**–**s** Histograms showing the contents of 4-HNE (**q**), MDA (**r**) and LPO (**s**). Data are presented as means ± s.d. ns, no significant difference; statistical significance levels: ^*^P < 0.05, ^**^P < 0.01 and ^***^P < 0.001.

To explore the role of Piezo1 in microvascular and skeletal muscle injury during ALIRI, we administered GsMTx4 intraperitoneally before I/R surgery in mice. LDI revealed that the blood flow density in the limbs treated with GsMTx4 was significantly increased compared to the I/R group (Fig. 2a, b). Conversely, the wet weight/dry weight ratio, an indicator of tissue edema, was markedly reduced (Fig. 2c). Immunofluorescence staining further demonstrated substantial upregulation of CD31 and CDH5 expression in vascular walls following GsMTx4 treatment (Fig. 2d–f). Consistent with these findings, the inhibition of Piezo1 activity reduced skeletal muscle fiber injury, as evidenced by Masson staining (Fig. 2g, h) and decreased infarct size as shown by TTC staining (Fig. 2i, j). Serum CK levels were also significantly lower in the GsMTx4-treated group compared to I/R controls (Fig. 2k). We also calculated the changes in ferroptosis levels after the administration of GsMTx4. After using GsMTx4, the mRNA and protein levels of ACSL4 decreased while the mRNA and protein levels of GPX4 increased (Fig. 2l–p). In addition, lipid peroxidation products (such as 4-HNE, MDA and LPO) were significantly reduced following GsMTx4 treatment (Fig. 2q–s). Collectively, these results indicate that Piezo1 inhibition attenuates both microvascular and skeletal muscle injury, concomitantly reducing ferroptosis during ALIRI.

Conversely, Yoda1 administration was employed to evaluate the impact of Piezo1 activation in ALIRI. LDI revealed reduced blood flow density in Yoda1-treated limbs compared to I/R controls (Supplementary Fig. 2a, b). The wet-to-dry weight ratio was significantly higher in the Yoda1 group (Supplementary Fig. 2c). Immunofluorescence analysis demonstrated decreased expression of CD31 and CDH5 (Supplementary Fig. 2d–f). Histological assessment revealed aggravated skeletal muscle fiber damage (Supplementary Fig. 2g, h) and increased infarct volume (Supplementary Fig. 2i,j). In addition, serum CK levels were elevated (Supplementary Fig. 2k). Both ferroptosis and lipid peroxidation products were significantly upregulated in the Yoda1-treated group relative to I/R controls (Supplementary Fig. 2l–s). Taken together, these findings provide strong evidence that Piezo1 activation exacerbates microvascular and skeletal muscle injury by promoting ferroptosis in ALIRI.

### MECs-specific *Piezo1* deletion alleviates ferroptosis and microvascular damage after ALIRI

**Fig. 3:**
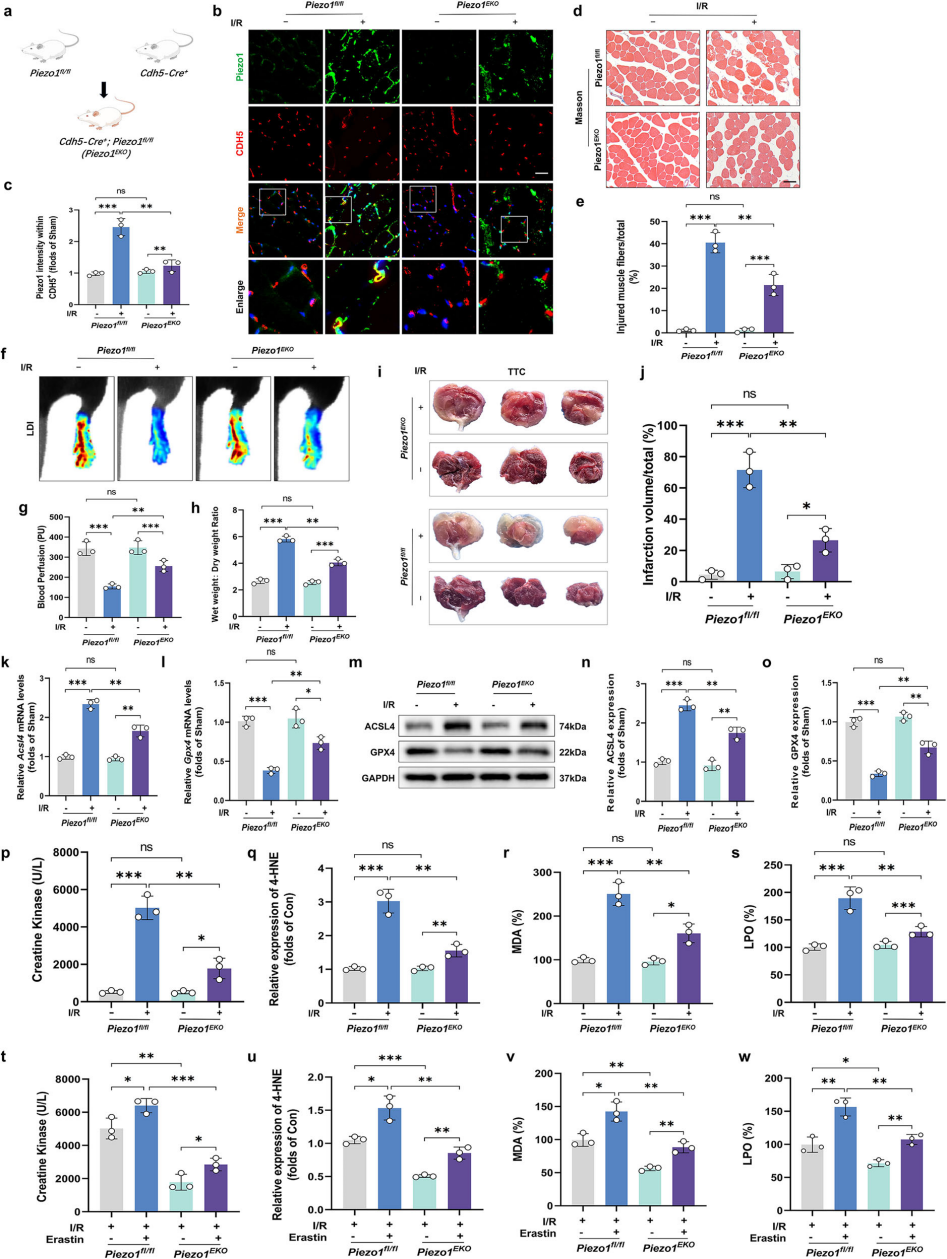
Specific deletion of Piezo1 in ECs mitigates ferroptosis and microvascular damage post-ALIRI. **a** A schematic depicting the breeding strategy to produce *Piezo1*^*EKO*^ mice, showing the crossing of *Piezo1*^*fl/fl*^ mice with *Cdh5-Cre* mice and the mating of heterozygous animals to generate embryos. Genetic testing outcomes for wild-type (WT), *Piezo1*^*EKO*^ and *Piezo1*^*fl/fl*^ mice are also illustrated. **b** Fluorescent images displaying co-localization of Piezo1 (green) and CDH5 (red). **c** Analysis of cells co-expressing Piezo1 and CDH5, alongside the proportion of total CDH5-positive cells. **d** Masson trichrome staining on skeletal muscle sections. Scale bars, 100 µm. **e** Calculation of the percentage of damaged fibers in skeletal muscle samples. **f** Blood perfusion in the hind limbs. **g** Histograms illustrating the intensity of blood flow. **h** The ratio of wet-to-dry weight used to assess tissue edema. **i**, TTC staining on sections to delineate areas of muscle damage. **j** Total volume quantification of gastrocnemius infarction. **k**, **l** Analysis of *Acsl4* (**k**) and *Gpx4* (**l**) relative expression. **m** Western blot images showing ACSL4 and GPX4 protein levels. **n**, **o** Quantitative analysis of ACSL4 (**n**) and GPX4 (**o**) proteins, normalized against GAPDH. **p** Serum levels of CK. **q**–**s** Histograms showing the contents of 4-HNE (**q**), MDA (**r**) and LPO (**s**). **t** Serum levels of CK. **u**–**w** Histograms showing the contents of 4-HNE (**u**), MDA (**v**) and LPO (**w**). Data are presented as means ± s.d. ns, no significant difference; statistical significance levels: ^*^P < 0.05, ^**^P < 0.01 and ^***^P < 0.001.

To investigate the role of Piezo1 in MECs and its impact on I/R injury, we generated a specialized mouse model known as *Piezo1*^*EKO*^. This model was created by controlling the expression of the murine *Piezo1* gene through the *Cdh5-Cre* recombinase system, as detailed in the schematic provided in Fig. 3a and Supplementary Fig. 3a. After developing these *Piezo1*^*EKO*^ mice, we proceeded to isolate MECs from adult mice and confirmed their identity through immunofluorescence techniques (Supplementary Fig. 3b). Our observations revealed that the *Piezo1*^*EKO*^ mice exhibited a marked reduction in Piezo1 fluorescence intensity on the vascular walls compared to the control *Piezo1*^*fl/fl*^ mice, particularly following I/R injury (Fig. 3b, c). To further understand the effects of Piezo1 deficiency on I/R injury, we evaluated various parameters in the mouse model. In *Piezo1*^*EKO*^ mice subjected to I/R injury, substantial improvements were observed compared to *Piezo1*^*fl/fl*^ controls. Specifically, limb perfusion, tissue edema, skeletal muscle fiber damage, infarct volume and serum CK levels were all markedly ameliorated (Fig. 3d–j, p). Moreover, both ferroptosis levels and lipid peroxidation products were significantly reduced in the *Piezo1*^*EKO*^ group relative to *Piezo1*^*fl/fl*^ mice following I/R (Fig. 3k–o, q–s). Interestingly, when the *Piezo1*^*EKO*^ mice were treated with erastin, a well-known inducer of ferroptosis, the protective effects of *Piezo1* deletion on skeletal muscle injury and ferroptosis were notably reversed (Fig. 3t–w). This finding indicates that while *Piezo1* deletion offers significant protection against skeletal muscle damage and reduces ferroptosis, the addition of ferroptosis inducers can negate these protective effects. In summary, our in vivo experiments demonstrate that Piezo1 activation in MECs exacerbates microvascular and tissue damage following ALIRI by promoting ferroptosis.

### Endothelial cPLA2 overexpression aggravates ALIRI-induced microvascular damage in *Piezo1*^*EKO*^ mice

**Fig. 4:**
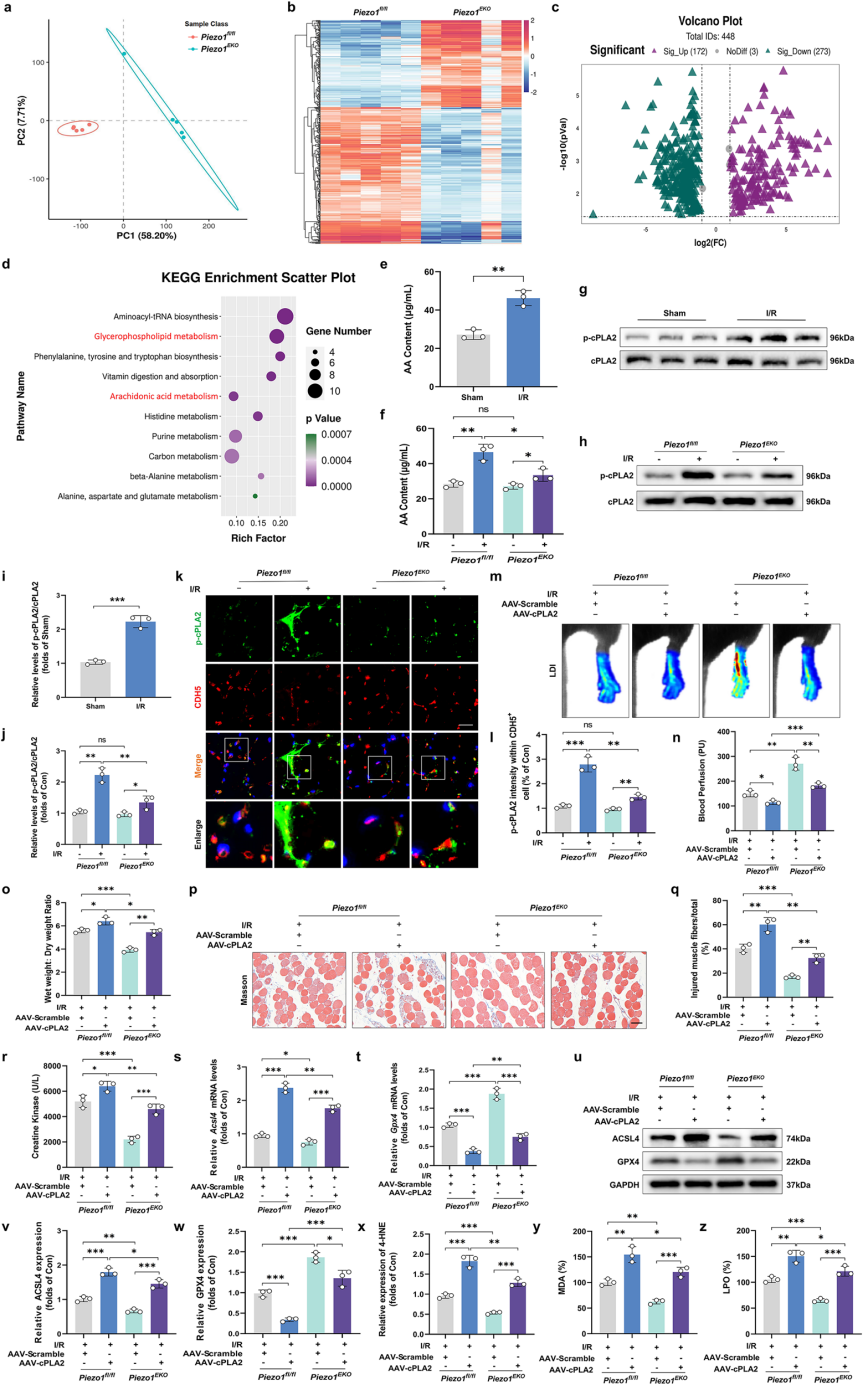
Impact of endothelial cPLA2 overexpression on microvascular damage in *Piezo1*^*EKO*^ mice post-ALIRI. **a** A PLS-DA plot illustrating the separation between *Piezo1*^*fl/fl*^ and *Piezo1*^*EKO*^ groups using positive ion mode UPLC–HDMS data. **b** A heat map displaying differential gene expression between *Piezo1*^*fl/fl*^ and *Piezo1*^*EKO*^ mice, with an intensity scale for expression levels. **c** A volcano plot highlighting genes that are upregulated (in red) and downregulated (in green) derived from the heat map in **b**. **d** A bubble plot representing the top ten KEGG pathways enriched from the differentially expressed genes. **e**, **f** Measurements of AA content in the sham and I/R (**e**) and *Piezo1fl/fl* and *Piezo1EKO* (**f**) groups. **g**, **h** Western blots showing p-cPLA2 and total cPLA2 protein levels in the sham and I/R (**g**) and *Piezo1fl/fl* and *Piezo1EKO* (**g**) groups. **i**, **j** Quantification of p-cPLA2 relative to total cPLA2 protein from the immunoblots in **g** (**i**) and h (**j**). **k** Immunofluorescent staining of skeletal muscle sections for p-cPLA2 and CDH5. Scale bars, 100 µm. **l** The count and percentage of double-positive p-cPLA2 and CDH5 cells relative to total CDH5-positive cells. **m** Blood perfusion in hind limbs. **n** A histogram depicting the signal intensity of blood flow. **o** The wet-to-dry weight ratio. **p** Masson’s trichrome staining of skeletal muscle sections for fibrous structure visualization. Scale bars, 100 µm. **q** Calculation of the damaged muscle fiber fraction. **r** Serum CK levels. **s**, **t** Analysis of *Acsl4* (**s**) and *Gpx4* (**t**) relative expression. **u** Western blot images showing ACSL4 and GPX4 protein levels. **v**, **w** Quantitative analysis of ACSL4 (**v**) and GPX4 (**w**) proteins, normalized against GAPDH. **x**–**z** Histograms showing the contents of 4-HNE (**x**), MDA (**y**) and LPO (**z**). Data are presented as means ± s.d. ns, no significant difference; statistical significance levels: ^*^P < 0.05, ^**^P < 0.01 and ^***^P < 0.001.

**Fig. 5:**
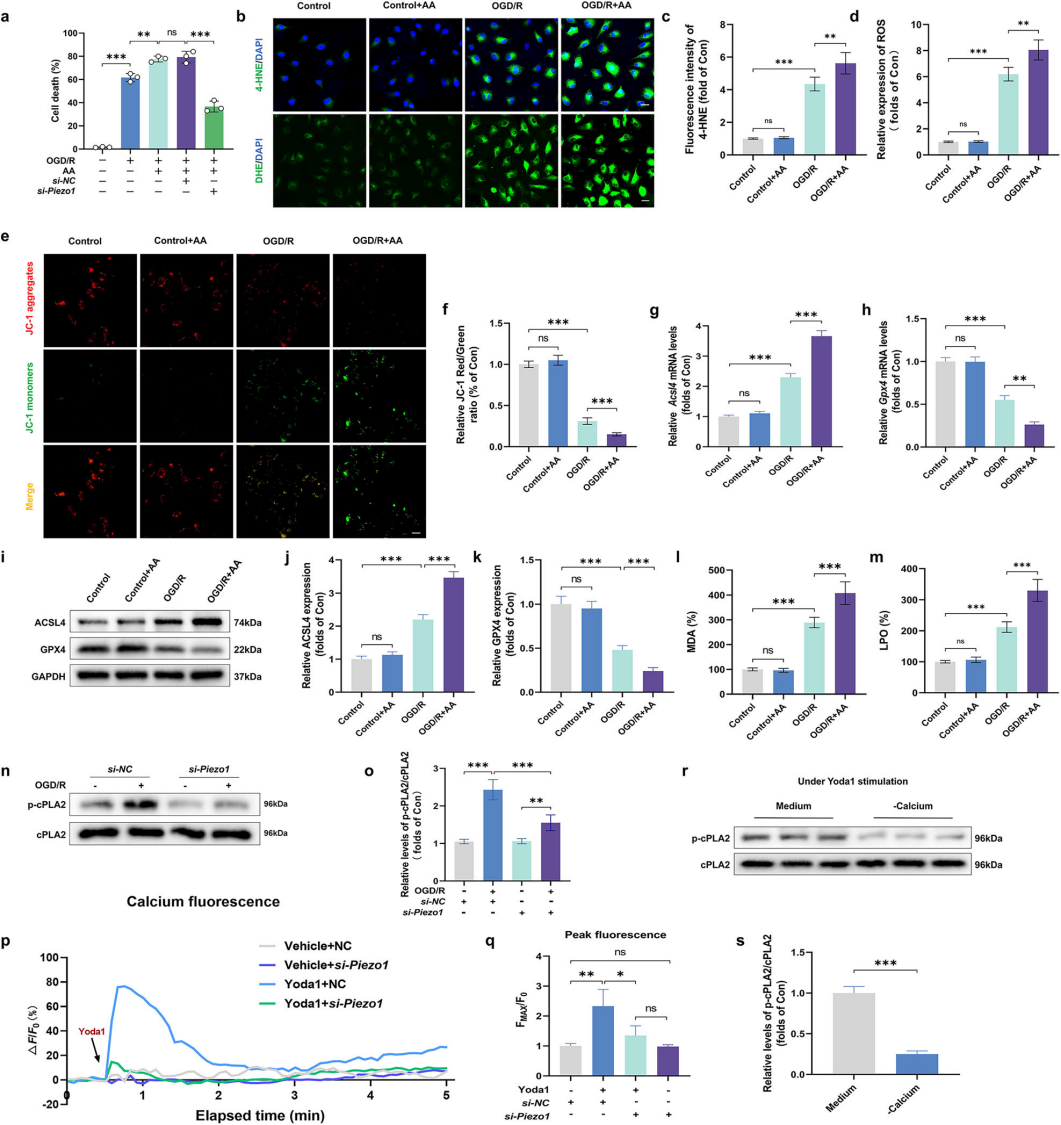
The impact of Piezo1 activation on ferroptosis in MECs through the Ca^2+^–cPLA2–AA pathway. **a** Analysis of cell death rates. **b** Immunofluorescence images showing staining for 4-HNE and DHE. Scale bars, 100 µm. **c** The average optical density of 4-HNE is shown. **d** Quantification of ROS levels. **e** JC-1 aggregate (red) and JC-1 monomer (green) staining of HUVECs. Scale bars, 100 µm. **f** Quantitation of aggregate/monomer fluorescence intensity ratio of JC-1 staining. **g**, **h** Analysis of *Acsl4* (**g**) and *Gpx4* (**h**) relative expression. **i** Western blot images showing ACSL4 and GPX4 protein levels. **j**, **k** Quantitative analysis of ACSL4 (**j**) and GPX4 (**k**) proteins, normalized against GAPDH. **l**, **m** Histograms showing the contents of MDA (**l**) and LPO (**m**). **n** Western blot images depicting phosphorylated and total cPLA2 protein levels. **o** The ratio of phosphorylated to total cPLA2. **p** The curve graph shows the relative amplitude of calcium signals (ΔF/F0) in HUVECs after Yoda1 and si-Piezo1 treatment. **q** Quantitative analysis of Ca²⁺ levels. **r** Western blots showing p-cPLA2 and total cPLA2 protein levels. **s** Quantification of p-cPLA2 relative to total cPLA2 protein. Data overview: displayed as means ± SD. Data are presented as means ± s.d. ns, no significant difference; statistical significance levels: ^*^P < 0.05, ^**^P < 0.01 and ^***^P < 0.001.

The precise mechanisms through which Piezo1 induces ferroptosis in MECs after I/R and contributes to subsequent microvascular and tissue damage remain unclear. AA is a crucial substrate involved in lipid peroxidation during ferroptosis^33^. Considering that arachidonate-metabolizing enzymes are known to be involved in the regulation of ferroptosis, we first investigated the differential metabolites in skeletal muscle tissues after I/R using untargeted metabolomic techniques. To achieve this, we analyzed the metabolic profiles of *Piezo1*^*EKO*^ and *Piezo1*^*fl/fl*^ mice. PLS-DA revealed distinct metabolic signatures between these two groups (Fig. 4a). Subsequent analyses provided robust evidence of significant variations, highlighting 172 genes with marked upregulation and 273 genes with pronounced downregulation (Fig. 4b, c). Further investigation into the pathways affected by these changes revealed that the differential metabolites were predominantly associated with pathways involved in glycerophospholipid metabolism and AA metabolism, as indicated by KEGG enrichment analysis (Fig. 4d). These results led us to hypothesize that Piezo1 exerts its influence on ferroptosis through modulation of AA metabolism. Consequently, we proceeded to assess the levels of AA metabolism-related biomarkers in the murine models to explore this potential mechanism further. To investigate the role of Piezo1 in modulating AA levels in skeletal muscle tissues, we first assessed AA content across the sham, I/R and *Piezo1*^*EKO*^ groups. Our results revealed a significant increase in AA levels in the I/R group compared to the sham controls, indicating a clear impact of I/R on AA metabolism (Fig. 4e). Notably, the *Piezo1* knockout effectively countered this increase, significantly reducing AA content in the I/R condition (Fig. 4f). Research has extensively demonstrated that cPLA2 plays a significant role in enhancing ferroptosis by facilitating the release of AA from membrane phospholipids, thereby promoting lipid peroxidation^14,34,35^. Next, we investigated whether there was a connection between Piezo1 and the expression and activity of cPLA2. We focused on evaluating the total amount of cPLA2 and its p-cPLA2, including the ratio of p-cPLA2 to total cPLA2, to better understand how Piezo1 influences cPLA2 activation. Western blot analysis showed that the absence of *Piezo1* substantially inhibited the phosphorylation of cPLA2 induced by I/R injury (Fig. 4g–j). Consistent with these findings, cell culture experiments demonstrated a significant reduction in cPLA2 phosphorylation in *si-Piezo1* cells (Fig. 5n, o). Furthermore, in *Piezo1*^*EKO*^ mice, the fluorescence intensity of p-cPLA2 on the vascular wall was elevated following I/R, yet it remained markedly lower compared to that in *Piezo1*^*fl/fl*^ mice (Fig. 4k, l). These results suggest that Piezo1 plays a critical role in facilitating the release of AA from membrane phospholipids through cPLA2 activation.

To further explore the involvement of cPLA2 in the context of microvascular and tissue damage post-I/R, we employed endothelium-specific AAV-cPLA2 to upregulate cPLA2 levels in MECs (Supplementary Fig. 7a). The administration of AAV-cPLA2 effectively increased the expression of total cPLA2 and phosphorylated cPLA2, thereby reversing the protective effects observed in Piezo1-deficient mice (Supplementary Fig. 4a–c). This was evidenced by a significant decrease in blood flow, an increase in skeletal muscle edema, exacerbated muscle fiber injury and elevated serum CK levels in the injured limbs (Fig. 4m–r). In addition, ferroptosis and lipid peroxidation levels were markedly elevated in the injured limbs following AAV-cPLA2 treatment (Fig. 4s–z). To further clarify the role of cPLA2, we also selected AACOCF3 (a selective cPLA2 inhibitor) to demonstrate effects of cPLA2 on the release of AA and ferroptosis. The results showed that inhibition of cPLA2 by AACOCF3 reduced AA release and significantly attenuated ferroptosis (Supplementary Fig. 4d–j). These observations collectively suggest that Piezo1 contributes to increased AA levels, thereby promoting ferroptosis and exacerbating tissue damage via cPLA2 activation.

### Activation of Piezo1 promotes ferroptosis in MECs via the Ca^2+^–cPLA2–AA axis

To further clarify the mechanism by which Piezo1 regulates the activity of cPLA2, we conducted a series of comprehensive experiments. Initially, we investigated the effect of exogenous AA on cell death in HUVECs using an OGD/R model. Our data, illustrated in Fig. 5a, demonstrated that AA treatment led to a substantial increase in cell death, but knocking down *Piezo1* significantly reduced cell death exacerbated by AA. This suggests that AA exacerbates cellular damage in the context of normal Piezo1 expression. Following this, we extended our investigation to explore whether AA administration could alter the progression of ferroptosis in HUVECs. We observed pronounced increases in ROS and lipid peroxidation products (4-HNE, MDA and LPO) exposed to OGD/R compared to untreated controls, and AA treatment further amplified these increases (Fig. 5b–d, l, m). JC-1 staining showed that AA exacerbated OGD/R-induced mitochondrial dysfunction (Fig. 5e, f). The mRNA and protein expression levels of ACSL4 and GPX4 further confirmed that the addition of excessive AA contribute significantly to the progression of ferroptosis (Fig. 5g–k).

Our investigations further extended to explore the intricate mechanisms underlying Piezo1’s role in Ca^2+^ influx regulation^36,37^. To determine whether Piezo1 activity is associated with Ca^2+^ influx, we stimulated HUVECs with Yoda1 and found a significant increase in the Ca^2+^ influx, whereas silencing *Piezo1* with siRNA under the same Yoda1 stimulation condition showed that the Ca^2+^ influx was obviously decreased (Fig. 5p, q), suggesting that Piezo1 plays an important role in regulating Ca^2+^ influx during OGD/R injury in vitro. To clarify the causal relationship between cPLA2 and Piezo1, calcium-free medium, which prevents the influx of Ca^2+^ was utilized to hinder the activation of Piezo1 induced by Yoda1. The results showed that phosphorylation of cPLA2 was blocked in calcium-free medium (Fig. 5r, s). To explore whether the process of cPLA2-promoted AA release is activated through Ca^2+^ flux, we administered EGTA-AM (a Ca^2+^-chelating agent) and assessed pharmacological outcomes. EGTA-AM treatment significantly reduced the activation of cPLA2 (Supplementary Fig. 5a, b). Besides, EGTA-AM reduced the levels of AA content, cell death rate, ROS production and lipid peroxidation (Supplementary Fig. 5c–h). Finally, we found that exogenous AA further enhanced the OGD/R-induced elevation of intracellular Ca^2+^, and this promoting effect was suppressed by GsMTx4 (Supplementary Fig. 5i, j). Those outcomes reveal that Piezo1 increases the content of AA to aggravate ferroptosis via promoting Ca^2+^-activated cPLA2 phosphorylation.

### Piezo1-mediated microvascular injury requires the ACSL4-associated ferroptosis pathway in ALIRI

**Fig. 6:**
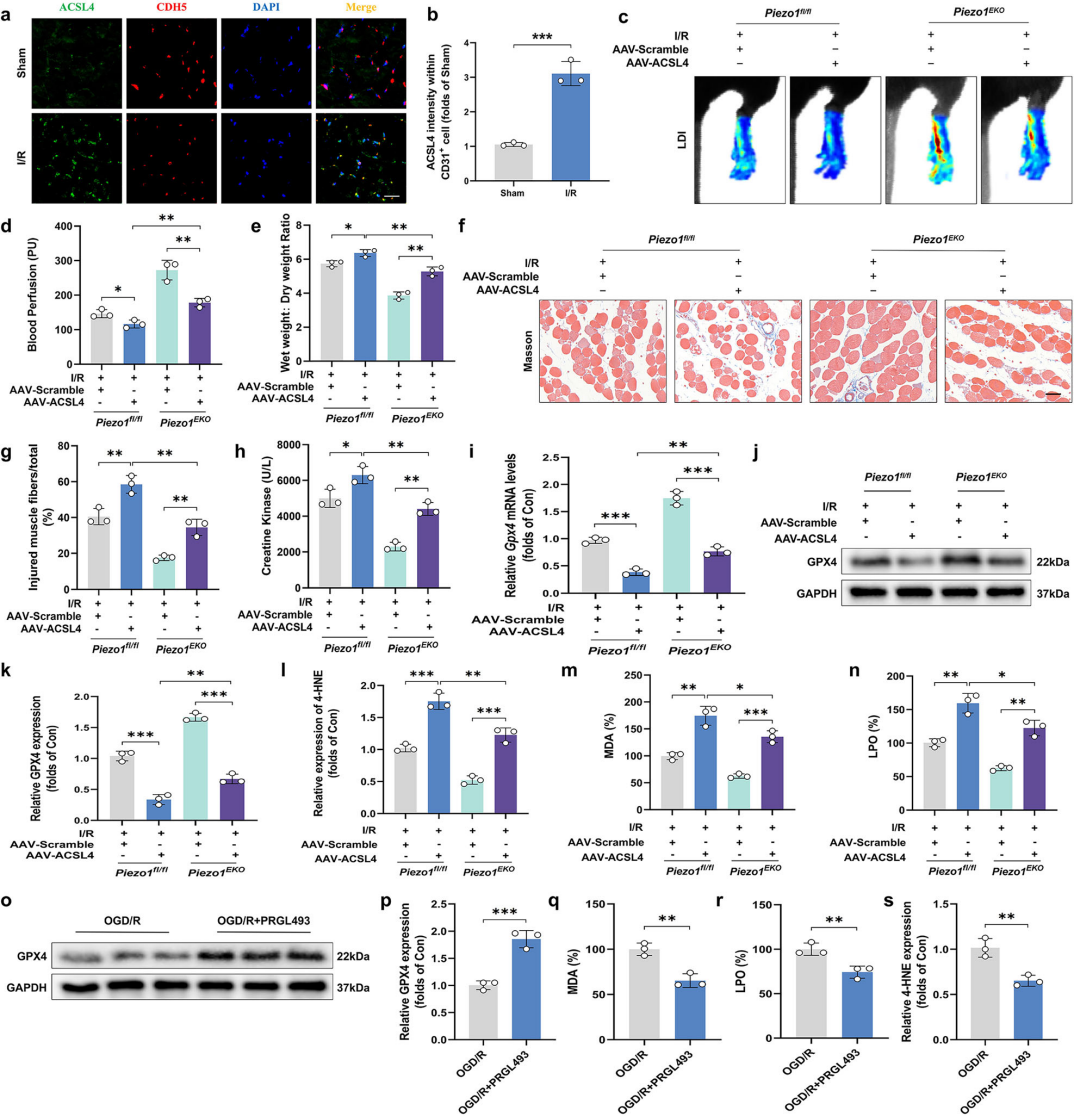
The role of ACSL4 in Piezo1-induced ferroptosis and microvascular damage during ALIRI. **a** Histological sections of skeletal muscle immunostaining for ACSL4 and CDH5, with nuclear identification via DAPI staining. Scale bars, 100 µm. **b** The proportions and counts of cells co-expressing ACSL4 and CDH5 relative to total CDH5-positive cells. **c** Blood perfusion in hind limbs. **d** A histogram representation of blood flow signal intensity. **e** The ratio of wet-to-dry weight to assess tissue hydration. **f** Masson’s trichrome staining applied to skeletal muscle sections. Scale bars, 100 µm. **g** Estimation of the proportion of damaged fibers within skeletal muscle. **h** Serum CK levels. **i** Analysis of *Gpx4* relative expression. **j** Western blot images showing GPX4 protein levels. **k** GPX4 protein quantification relative to GAPDH. **l**–**n** Histograms showing the contents of 4-HNE (**l**), MDA (**m**) and LPO (**n**). **o** Western blot images showing GPX4 protein levels in HUVECs. **p** GPX4 protein quantification relative to GAPDH. **q**–**s** Histograms showing the contents of 4-HNE (**q**), MDA (**r**) and LPO (**s**). Data are presented as means ± s.d. ns, no significant difference; statistical significance levels: ^*^P < 0.05, ^**^P < 0.01 and ^***^P < 0.001.

ACSL4 is recognized as a pivotal enzyme that not only promotes the biosynthesis and remodeling of phosphatidylethanolamine (PE) but also catalyzes lipid peroxidation, critical to the ferroptosis process^38^. This enzyme produces AA-CoA, which is vital for the acylation of lysophospholipids. Consequently, ACSL4 plays a significant role in generating a pool of PUFAs that serve as targets for lipid peroxidation^39^. ACSL4 protein levels in the limbs of I/R injury model were significantly higher compared to the sham operated control group (Fig. 2n, o and Supplementary Fig. 2n, o). Furthermore, the fluorescence intensity of skeletal muscle tissues of the I/R group was significantly higher than that of the sham group in the vascular walls (Fig. 6a, b). To further elucidate the role of ACSL4 in microvascular and tissue damage resulting from I/R, we utilized an AAV vector designed for endothelial cell (EC)-specific expression of ACSL4. Administration of AAV-ACSL4 successfully increased ACSL4 levels in MECs (Supplementary Fig. 7b). This intervention significantly negated the protective effects of *Piezo1* deficiency, as evidenced by a reduction in blood flow, exacerbation of skeletal muscle edema, increased skeletal muscle fiber damage and elevated serum levels of CK in the injured limbs (Fig. 6c–h). Furthermore, the group receiving AAV-ACSL4 treatment displayed considerably higher levels of ferroptosis and lipid peroxidation products (Fig. 6i–n). In addition, cell experiments with PRGL493 (a selective inhibitor of ACSL4) demonstrated that ferroptosis level was significantly reduced upon ACSL4 inhibition (Fig. 6o–s). These observations suggest that Piezo1 enhances lipid peroxidation-mediated ferroptosis through the activation and involvement of ACSL4.

### Endothelial PKCβII overexpression promotes ACSL4-mediated lipid peroxidation and aggravates ALIRI-induced MECs ferroptosis-related microvascular damage in *Piezo1*^*EKO*^ mice

**Fig. 7:**
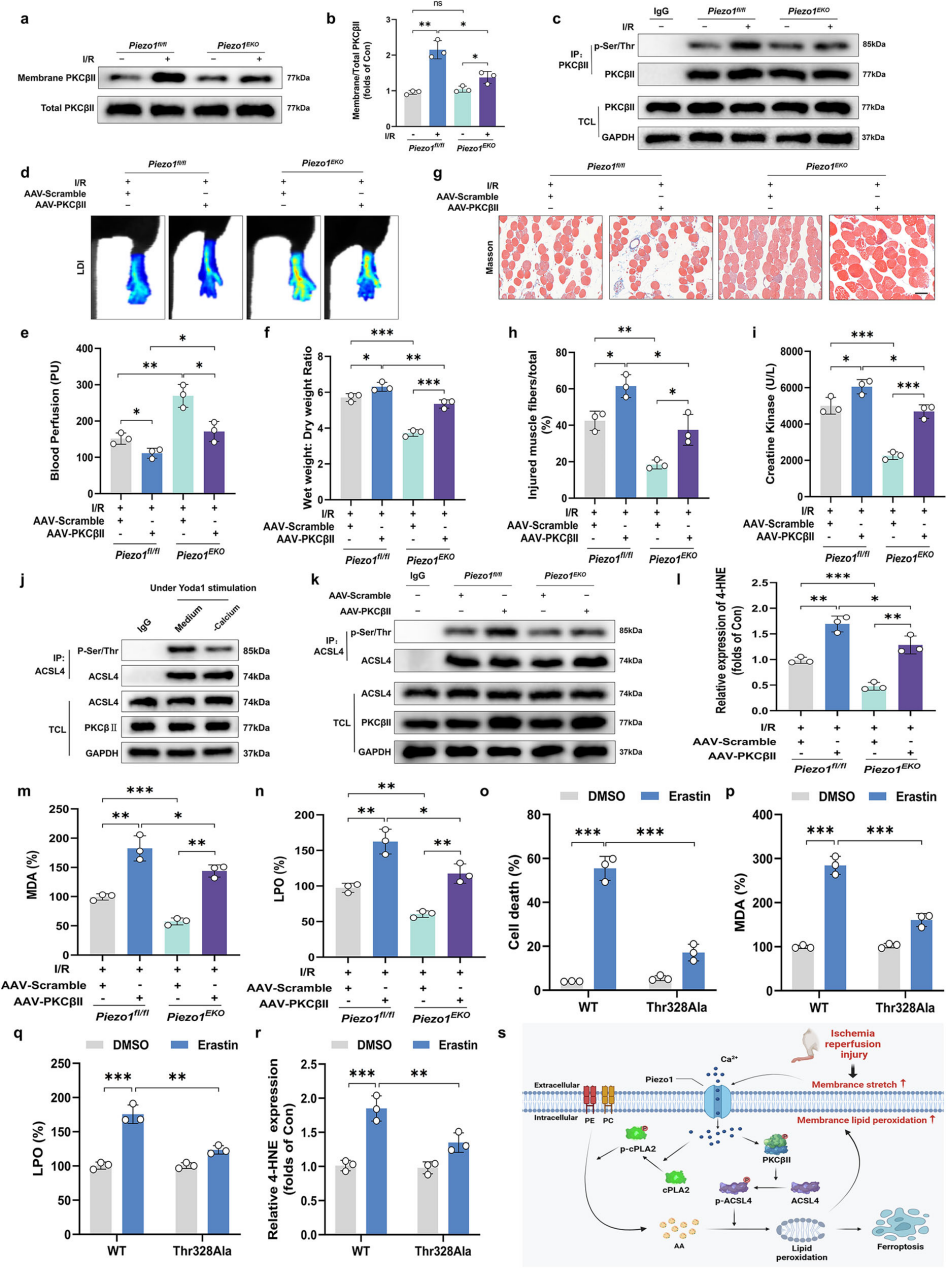
Influence of endothelial PKCβII overexpression on ACSL4-driven lipid peroxidation and enhanced ferroptosis-related microvascular damage in *Piezo1*^*EKO*^ mice post-ALIRI. **a** Western blot images showing the expression levels of membrane-bound and total PKCβII. **b** Quantitative assessment of the ratio of membrane-bound to total PKCβII. **c** Immunoblots depicting phosphorylation of Ser/Thr residues and PKCβII levels. **d** Blood perfusion in the hind limbs. **e** A histogram of blood flow signal intensity measurements. **f** Calculation of tissue hydration through the wet-to-dry weight ratio. **g** Masson’s trichrome staining on sections of skeletal muscle. Scale bars, 100 µm. **h** An analysis of the proportion of damaged muscle fibers. **i** CK levels as markers of muscle damage. **j**, **k** Western blots showing phosphorylation of Ser/Thr residues (**j**) and ACSL4 protein expression (**k**). **l**–**n** Histograms showing the contents of 4-HNE (**l**), MDA (**m**) and LPO (**n**). **o** The proportion of cell death. **p**–**r** Histograms showing the contents of MDA (**p**), LPO (**q**) and 4-HNE (**r**). **s** A schematic illustrating the proposed molecular mechanisms that delineate the roles of Piezo1-Ca²⁺ signaling, cPLA2, ACSL4, AA, lipid peroxidation and ferroptosis in the pathophysiology of MEC injury induced by ALIRI. Data overview: presented as means ± SD. ns, no significant difference; statistical significance levels ^*^P < 0.05, ^**^P < 0.01 and ^***^P < 0.001.

A recent study shows that the PKCβII–ACSL4 signaling pathway is important in the regulation of ferroptosis by increasing lipid peroxidation to a level that is toxic to cells^16^. In particular, this axis functions via the activation of PKCβII, which belongs to the conventional PKC subgroup. PKCβII has a C2 domain that can bind Ca^2+^, which is critical for its activation^40^. This domain is sensitive to the changes in intracellular Ca^2+^ levels, thus allowing PKCβII to be involved in the rapid and excessive lipid peroxidation, which is a hallmark of ferroptosis^41^. Together, our data indicate that Piezo1-mediated Ca^2+^ influx leads to activation of the PKCβII–ACSL4 signaling cascade, which is essential for promoting lipid peroxidation to catastrophic levels that result in ferroptosis. The major finding of the present study was that Piezo1 significantly increased the translocation of PKCβII to the cell membrane as evidenced by Fig. 7a, b. Moreover, immunoprecipitation followed by western blotting demonstrated that the absence of *Piezo1* leads to a significant reduction in PKCβII phosphorylation induced by I/R injury, as depicted in Fig. 7c. To further explore this mechanism, we assessed microvascular and tissue damage in *Piezo1*^*EKO*^ and *Piezo1*^*fl/fl*^ mice subjected to either AAV-PKCβII or AAV-Scramble control treatments. By employing an endothelium-specific AAV-PKCβII vector, we were able to effectively upregulate PKCβII levels in MECs (Supplementary Fig. 7c). The administration of AAV-PKCβII notably reversed the protective effects of *Piezo1* deficiency on microvascular and tissue damage (Fig. 7d–i). To elucidate whether ACSL4-induced lipid peroxidation is influenced by the Piezo1–Ca^2^^+^–PKCβII pathway, we examined the effects of Piezo1 and PKCβII on ACSL4 activation. Our immunoprecipitation and western blot analysis confirmed that the calcium-free culture medium significantly inhibited Yoda1-induced ACSL4 phosphorylation (Fig. 7j). The use of AVV-PKCβII demonstrated that PKCβII facilitates the phosphorylation of ACSL4, which represents a crucial step in ferroptosis induction (Fig. 7k). Furthermore, the AAV-PKCβII treatment group exhibited significantly elevated levels of lipid peroxidation products (Fig. 7l–n). To further clarify the crucial role of ACSL4 activation in ferroptosis, we constructed the ACSL4–Thr328Ala mutant^16^. As expected, erastin-induced ferroptosis was dramatically reduced in cells expressing the ACSL4–Thr328Ala mutant (Fig. 7o–r). Overall, these findings demonstrate that PKCβII drives ferroptosis by phosphorylating and activating ACSL4, and that Piezo1 exacerbates ferroptosis following I/R injury through the PKCβII–ACSL4 signaling pathway.

## Discussion

Acute lower limb ischemia is a prevalent and urgent peripheral vascular condition characterized by an abrupt and significant decrease in blood supply to the lower extremities. This severe reduction in blood flow often leads to symptoms such as intermittent claudication, which can progress to irreversible limb necrosis if not promptly addressed. In the clinical management of acute lower limb ischemia, the restoration of blood flow in the ischemic limb is of paramount importance^42^. Despite the critical nature of this intervention, challenges persist, particularly concerning the injury sustained by the microvasculature. The existing literature also reveals that the microcirculatory perfusion during the reperfusion phase is often limited and such factors might aggravate limb ischemia^43^. At the time of reperfusion, MECs are more sensitive to the damaging factors in the blood circulation, making them affected by I/R injury at an early stage^44^. The initial damage caused leads to the death of MECs, which in turn results in increased tissue injury and poor recovery. Prior research has highlighted the death of MECs as one of the critical mediators of microvascular injury after I/R. Furthermore, the pharmacological interventions that have been attempted to minimize the death of MECs have been observed to considerably decrease microvascular damage and enhance limb viability after ALIRI^6,32^. Presently, therapeutic agents directed at ECs are widely used in the management of cardiovascular diseases including myocardial infarction and arteriosclerotic occlusive disease, which shows the relevance of such approaches in the management of patients^45,46^. Clinical investigations have indicated that early restoration of blood circulation after an ischemic attack of limbs generally leads to a high degree of microvascular injury and congestion. This may cause increased arteriovenous shunting and worsen tissue edema as seen in wounded skin after burn induced injury^3^. This chain of events increases the pressure of the tissue and may lead to the appearance of ACS. The relationship between intrafascial pressure increase and tissue injury in the context of ALIRI is critical in the study of the pathogenesis and the course of ACS. Therefore, it is necessary to explore the relations between intrafascial pressure and MECs damage to develop an understanding of the process. By understanding the mechanisms, it may become possible to find ways of breaking the cycle of ischemia and edema, which leads to preventing further ischemia. The above approaches could therefore present viable means of early identification and management of ACS thus enhancing the prognosis in patients who present with ALIRI.

Ferroptosis, a form of cell death that involves lipid peroxidation, has been identified as a major form of cell death in I/R injury that is mainly caused by ROS^47^. This process involves a series of enzyme-mediated reactions that culminates in the generation of lipid peroxidation products^48^, which can enhance the membrane damage and tissue swelling. Current research indicates that AA generates proinflammatory mediators through the lipoxygenase/cyclooxygenase (LOX/COX) pathways. However, the core mechanism leading to ferroptosis is the direct oxidation of AA (and other PUFA) by the LOX enzyme, rather than through downstream products of the LOX/COX pathways^49^. The proinflammatory mediators produced by the LOX/COX pathway (prostaglandins and leukotrienes) do not directly cause ferroptosis. They may indirectly create conditions conducive to ferroptosis through exacerbating the inflammatory response and OS, but the executive mechanism of ferroptosis remains the LOX-mediated, iron-dependent lipid peroxidation process. Even though the exact pathways of ferroptosis in ALIRI are not yet known, we found that lipid peroxidation levels were elevated after ALIRI. Also, Fer-1, an inhibitor of ferroptosis, was able to decrease lipid peroxidation and reduce ferroptosis, thereby ameliorating both microvascular and skeletal muscle injury. On the basis of these observations, it is obvious that modulating ferroptosis linked to lipid peroxidation in MECs may be a potential therapeutic strategy for microvascular dysfunction in ALIRI. This approach may hold the promise of halting the pathological process of ALIRI and therefore enhance patient outcomes by reducing microvascular damage.

In ferroptosis, lipid metabolism is initiated by the release of bioactive lipids, which are primarily regulated by three distinct types of phospholipase A2 (PLA2): Ca^2+^-independent PLA2 (iPLA2), cPLA2 and secretory PLA2 (ref. ^50^). Among these, cPLA2 exhibits a particular substrate specificity for AA, which is the most prevalent and biologically active PUFAs in the human body. The activated cPLA2 hydrolyzes AA from the sn-2 position of glycerophospholipids^51^. This enzymatic action results in elevated cytosolic levels of free AA^34,52^, which subsequently accelerates lipid peroxidation processes and promotes ferroptosis, especially following the activation of ACSL4. The cPLA2 is a Ca^2+^-dependent enzyme that is activated by increased intracellular Ca^2+^ concentrations, leading to autophosphorylation at serine residues 505 and 515 (ref. ^53^). Once liberated, free PUFAs act as substrates for the synthesis of lipid signaling transduction mediators. These mediators are esterified into membrane phospholipids and subsequently oxidized as ferroptosis signals^54,55^. In eukaryotic cells, AA, the primary substrate for ACSL4, is converted into acyl-CoA by the activated ACSL4. This acyl-CoA is then incorporated into membrane phospholipids by lysophosphatidylcholine acyltransferase 3 (LPCAT3), which enhances the cell’s susceptibility to ferroptosis^11,56^. Consistent with these observations, our study demonstrates that there is a marked increase in both the expression and activity of cPLA2 in MECs after I/R injury. Furthermore, the overexpression of cPLA2 exacerbates ACSL4-dependent lipid peroxidation in MECs, leading to severe cellular and tissue damage.

Previous investigations have established that PKCβII, which is a component of the classical PKC family, possesses a Ca^2+^-binding domain situated in its C2 region^40^. This domain is responsive to elevated intracellular Ca^2+^ levels, leading to the activation of PKCβII and subsequent stimulation of ACSL4 (refs. ^16,41^). Our recent research has provided evidence that, within the framework of ALIRI, the activation of the PKCβII–ACSL4 signaling cascade plays a crucial role in intensifying lipid peroxidation level of free AA. This intensification promotes ferroptosis specifically in MECs. These underscores the important role of ACSL4 activation in mediating AA lipid peroxidation during the ferroptosis process, indicating that further detailed studies are warranted to fully understand the mechanisms driving this interaction and its implications in ALIRI.

Recent research has increasingly demonstrated that an excessive buildup of lipid peroxidation products predominantly occurs within cellular membranes. This accumulation leads to heightened membrane tension, which in turn activates a range of mechanosensitive ion channels, including Piezo1. The activation of Piezo1 has been linked to an exacerbation of cellular damage^57^. Piezo1 is known for its response to mechanical stimuli such as hydrostatic pressure, fluid shear stress and changes in membrane tension^19,25^. When Piezo1 is activated, it induces a significant influx of Ca^2+^ ions, resulting in an intracellular Ca^2+^ overload. This overload then triggers a cascade of Ca^2+^-dependent signaling pathways^58,59^. Despite these findings, there remains a gap in the research regarding the specific role of Piezo1 in mediating lipid peroxidation and ferroptosis in the context of I/R injury.

The mechanisms underlying increased Piezo1 expression in ECs are multifactorial, involving both physiological and pathological stimuli and complex molecular regulatory networks. Mechanical stimuli, such as fluid shear stress, traction/tension and matrix stiffness, trigger the expression of Piezo1 by activating Piezo1 and downstream signaling pathways, thereby upregulating the expression of Piezo1^60,61^. Chemical factors and cytokines, including inflammatory factors, can directly or indirectly promote the expression of Piezo1 by activating the nuclear factor kappa-light-chain-enhancer of activated B cells (NF-κB) signaling pathway^60^, and vasoactive substances upregulate the expression of Piezo1 by activating downstream kinases such as mitogen-activated protein kinase (MAPK) and activator protein-1 transcription factors^62^. In addition, under ischemic and hypoxic conditions, the activation of hypoxia-inducible factor 1-alpha (HIF-1α) can upregulate the expression of transforming growth factor beta 1 (TGFβ1), promote the Piezo1-mediated endothelial–mesenchymal transition and establishes a positive feedback loop with Piezo1 to maintain its expression^63^.

Piezo1 is mainly expressed in nonsensory tissues and mediates mechanical responses of various cell types, including ECs, astrocytes, macrophages, fibroblasts and so on. Extensive studies have demonstrated that Piezo1 activation in macrophages and fibroblasts induces Ca^2+^ influx, driving proinflammatory signaling pathways and the release of inflammatory factors (interleukin-1 beta (IL-1β), interleukin-6 (IL-6), interleukin-8 (IL-8) and tumor necrosis factor (TNF)) and chemokines, thereby regulating the inflammatory response of immune cells^64–67^. The Piezo1 activation in macrophage also regulates migration and phenotypic transformation, contributing to local vascular remodeling^68^. Meanwhile, the Piezo1 activation in fibroblasts triggers Ca^2+^ influx and YAP/TAZ signaling pathways, promoting myofibroblasts differentiation and excessive extracellular matrix secretion, thereby driving fibrosis and tissue stiffening^69^. Macrophages and fibroblasts create a positive feedback loop through inflammatory mediators and the stiffened microenvironment, perpetuating inflammation and fibrosis^70^. Interestingly, Piezo1 also regulates the interaction and function between neurons and astrocytes^71^. Thus, we hypothesize that the Piezo1 activation in other types of cells can indirectly affect ECs, contributing to microvascular and skeletal muscle injury after ALIRI. However, the precise stages and mechanisms of these intercellular interactions mediated by Piezo1 remain to be further elucidated.

To our knowledge, our study is pioneering in demonstrating that activation of the Piezo1–Ca^2+^ channel complex in MECs following ALIRI substantially influences AA metabolism. This metabolic alteration, in turn, triggers ACSL4-mediated lipid peroxidation. Nonetheless, the mechanisms through which Piezo1 modulates the influx of various ions remain complex and not fully elucidated. Although we employed metabolomics to analyze tissue-level metabolic changes, this approach could not specifically resolve metabolite alterations within ECs. While the observed metabolic shifts after endothelial-specific *Piezo1* knockout mainly occur within ECs, these metabolites may also influence other cell types. In future studies, metabolomics analyses based on primary skeletal muscle MECs will help reduce confounding effects from other cell populations.

The role of Piezo1 extends well beyond mere ion channel functions to encompass a wide range of physiological and pathological processes, including but not limited to the development of lymphatic vessels, axon growth, vascular remodeling, immune responses and blood pressure regulation. Despite these diverse functions, the pharmacological targeting of Piezo1 is still in its nascent stages, with a limited number of specific agonists and antagonists currently available. The prevalence of Piezo1 in different cell types, coupled with the broad, nonspecific effects of most existing Piezo1 antagonists, presents a substantial obstacle to the development of precise and safe therapeutic interventions. For instance, clinical applications of Piezo1 modulators face challenges such as poor solubility, low stability and inadequate affinity of both the activators (for example, Yoda1) and blockers (for example, GsMTx4)^72^. Furthermore, the large molecular size of Piezo1 complicates the production of associated AAV vectors. The high costs, delayed onset of action, challenges in maintaining effective vector concentrations, genetic toxicity and potential carcinogenicity associated with AAV gene therapy further limit its practical clinical use^73^. By contrast, low-intensity pulsed ultrasound (LIPUS) presents a promising alternative. This therapeutic modality, characterized by its low intensity and pulsed wave output, offers several advantages including cost-effectiveness, noninvasiveness, absence of infection risks, straightforward treatment protocols and fewer complications. LIPUS has demonstrated protective effects across various models of I/R injury^74^, and research indicates that it significantly reduces Piezo1 expression levels in targeted cells, thereby aiding in the recovery of both sports injuries and neural damage^75,76^. Compared to traditional surgical methods, LIPUS provides an economical, effective and minimally invasive rehabilitation strategy, which has considerable clinical relevance and potential for improving outcomes in patients with ALIRI. However, it is essential to recognize that the effects of LIPUS on Piezo1 channels can vary depending on the specific parameters used. Different LIPUS settings may either activate or inhibit Piezo1 channels, underscoring the need for precise tuning of frequency and intensity to maximize therapeutic benefits. By optimizing these parameters, LIPUS treatment could potentially reduce microvascular damage and facilitate limb function recovery following ALIRI through targeted modulation of Piezo1 channel activity.

In conclusion, our research presents pioneering evidence establishing a connection between mechanical signals and ALIRI. This study elucidates the complex interplay between tissue swelling and MECs damage within the ALIRI context. Our findings reveal that AA lipid peroxidation is a primary mechanism driving both tissue edema and cellular injury in ALIRI. Specifically, the activation of cPLA2 facilitates the release of AA from cellular membranes, which subsequently intensifies lipid peroxidation reactions through the activation of ACSL4. This cascade of events ultimately exacerbates the extent of damage. Furthermore, our study demonstrates that, under I/R conditions, Piezo1 channels become activated, leading to a significant influx of Ca^2+^. This Ca^2+^ influx activates cPLA2, further promoting AA release. In addition, the elevated Ca^2+^ levels activate the PKCβII–ACSL4 signaling axis, which amplifies the lipid peroxidation level of AA, thereby contributing to the progression of ferroptosis (Fig. 7s). Interestingly, the Piezo1/Ca^2+^–cPLA2 pathway and the Piezo1/Ca^2+^–PKCβII–ACSL4 pathway can influence each other (Supplementary Fig. 6a–d). These data suggest that targeting Piezo1 represents a promising strategy for early intervention aimed at disrupting the harmful ‘ischemia–edema–ischemia’ cycle. By doing so, it may be possible to mitigate microvascular and tissue damage, potentially halting the progression of ACS. On the basis of these findings, future research should focus on validating Piezo1 as a novel therapeutic target for ALIRI. Such validation could provide a critical foundation for subsequent clinical trials, thereby advancing the development of effective treatments for ALIRI.

### Ethics approval and consent to participate

The entire experimental methods and procedures followed the Animal Care and Use Committees guidelines approved by the Animal Research Ethics Committee of Wenzhou Medical University (wydw 2023-0248).

## Availability of data and materials

All data needed to evaluate the conclusions in the paper are presented in the paper and/or in the Supplementary Materials. Additional data related to this paper may be requested from the authors.

## Supplementary information

Supplementary Information
