# Supplementary material for: Piezo1 activation in endothelial cells aggravates microvascular ischemia–reperfusion injury in limbs by enhancing ferroptosis
Source: Exp Mol Med. 2026 Jan 9;58(1):143–60. doi: 10.1038/s12276-025-01616-9 (PMC12868677; doi:10.1038/s12276-025-01616-9)
Supplement: Supplementary file 1 — Supplementary Information [file 12276_2025_1616_MOESM1_ESM.pdf]

Supplementary Information

for

Piezo1 activation in endothelial cells aggravates microvascular ischemia reperfusion injury in limbs by enhancing ferroptosis

Fanfeng Chen *et. al*

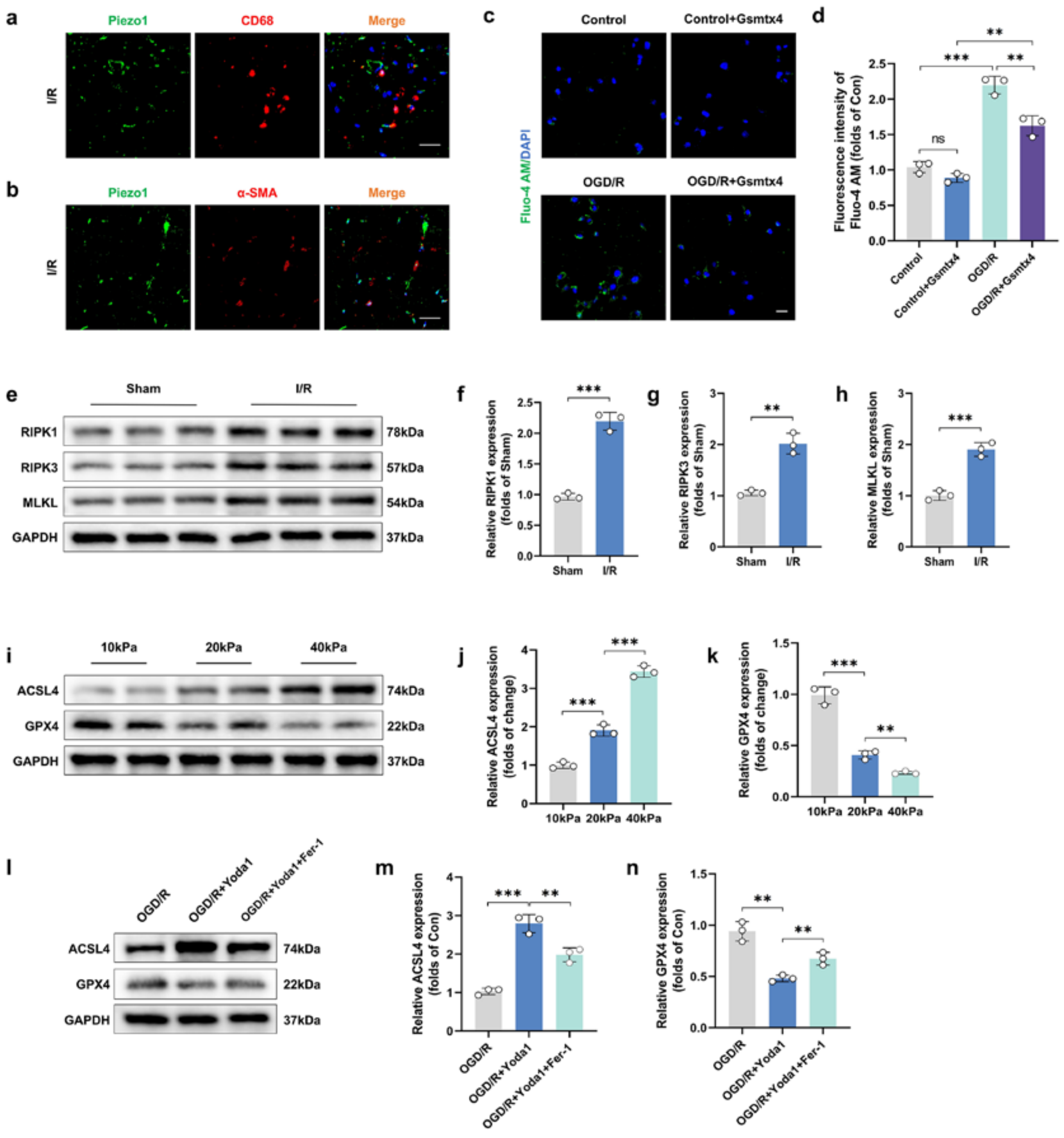

Supplementary Figure 1. Regulation and Impact of Piezo1 in ALIRI with a Focus on Ferroptosis as the Predominant Cell Death Mode. (a) Histological sections of skeletal muscle from mice, immunostaining for Piezo1 and CD68. Scale bars represent 100 µm. (b) Histological sections of skeletal muscle from mice, immunostaining for Piezo1 and α-SMA. Scale bars represent 100 µm. (c) Immunofluorescence images showing staining for Fluo-4 AM, with DAPI highlighting nuclei. Scale bars at 100 µm. (d) The average optical density of Fluo-4 AM is presented. (e) Western blot images showing RIPK1, RIPK3 and MLKL protein levels. (f-h) Quantitative analysis of

RIPK1, RIPK3 and MLKL proteins, normalized against GAPDH. (i) Representative immunoblots of ACSL4 and GPX4 expression in HUVECs seeded on 10, 20, and 40 kPa polyacrylamide gels. (j-k) Quantitative analysis of ACSL4 and GPX4 proteins, normalized against GAPDH. (l) Western blot images showing ACSL4 and GPX4 protein levels. (m-n) Quantitative analysis of ACSL4 and GPX4 proteins, normalized against GAPDH. Data overview: presented as means ± SD. Significance notation: "ns" indicates no significant difference; Statistical significance levels: **(P < 0.01) and ***(P < 0.001).

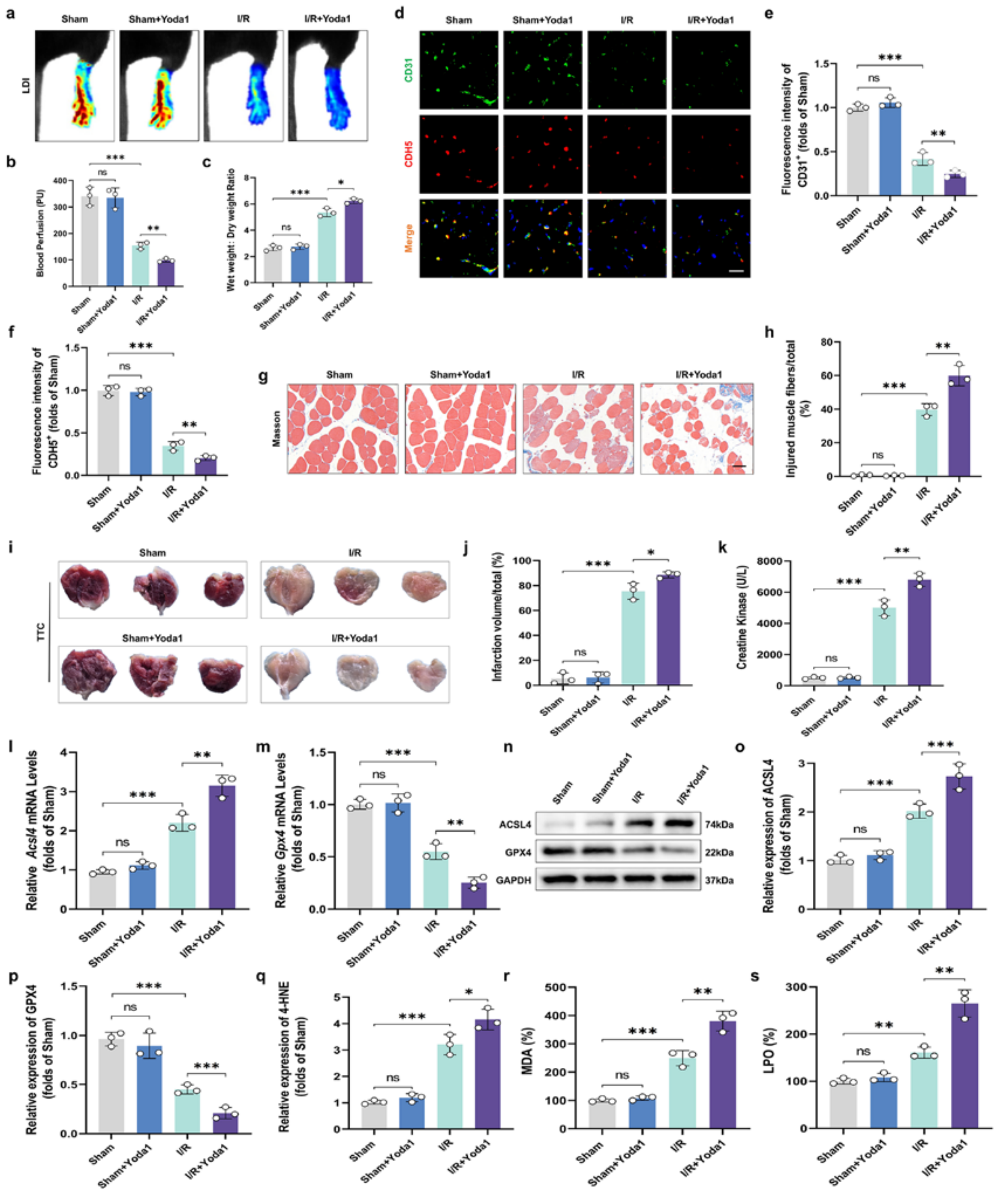

Supplementary Figure 2. Enhanced Piezo1 Activity Aggravates ALIRI-Related Ferroptosis and Microvascular Injury. (a) Blood perfusion in the hind limbs. (b) Histogram depicting the intensity of blood flow signals. (c) Measurement of tissue edema through the wet-to-dry weight ratio. (d) Histological sections of skeletal muscle from mice, immunostaining for CD31 and CDH5. Scale bars represent 100 μm. (e-f) Quantification of mean optical density values of CD31 and CDH5. (g)

Masson's trichrome staining of skeletal muscle sections to highlight muscle fiber architecture. Scale bars: 100 μm. (h) Evaluation of the proportion of damaged fibers in skeletal muscle. (i) TTC staining employed to assess the extent of muscle damage in skeletal sections. (j) Total volume of infarction in the gastrocnemius quantified. (k) Serum CK levels. (l-m) Analysis of *Acsl4* and *Gpx4* relative expression. (n) Western blot images showing ACSL4 and GPX4 protein levels. (o-p) Quantitative analysis of ACSL4 and GPX4 proteins, normalized against GAPDH. (q-s) Histograms showing the contents of 4-HNE, MDA and LPO. Data overview: presented as means ± SD. Significance notation: "ns" indicates no significant difference; Statistical significance levels: *($P < 0.05$), **($P < 0.01$) and ***($P < 0.001$).

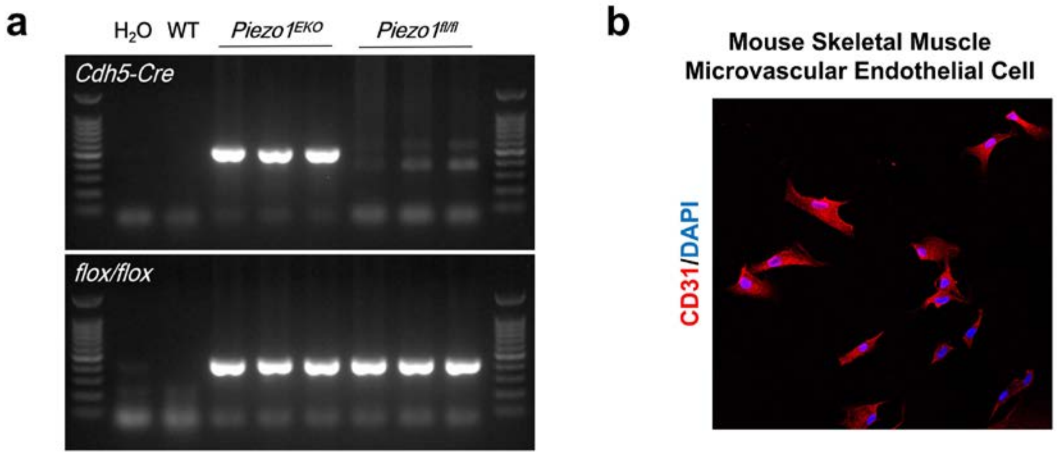

Supplementary Figure 3. Piezo1 expression and validation of knockdown in ECs. (a) Genetic testing outcomes for wild type (WT), *Piezo1*^{EKO}, and *Piezo1*$^{fl/fl}$ mice are illustrated. (b) Immunofluorescence imaging of CD31 (red) in MECs isolated form mouse skeletal muscle, with scale bars of 100 μm.

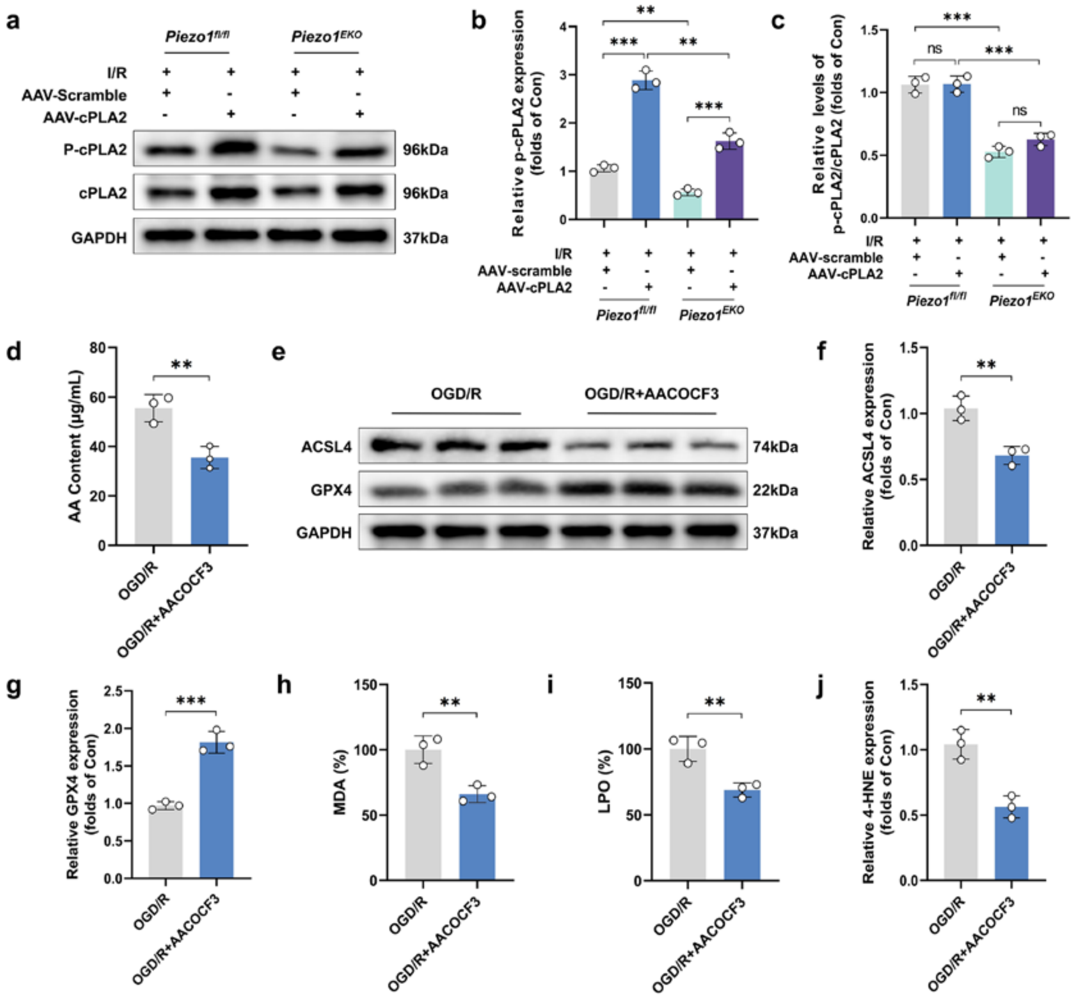

Supplementary Figure 4. Impact of Endothelial cPLA2 Overexpression on Microvascular Damage in *Piezo1*^{EKO} Mice Post-ALIRI. (a) Western blots showing p-cPLA2 and total cPLA2 protein levels. (b) Quantitative analysis of p-cPLA2. (c) Quantification of p-cPLA2 relative to total cPLA2 protein from the immunoblots. (d) Measurements of AA content. (e) Western blot images showing ACSL4 and GPX4 protein levels. (f-g) Quantitative analysis of ACSL4 and GPX4 proteins, normalized against GAPDH. (h-j) Histograms showing the contents of 4-HNE, MDA and LPO. Data overview: presented as means ± SD. Significance notation: "ns" indicates no significant difference; Statistical significance levels: **(P < 0.01) and ***(P < 0.001).

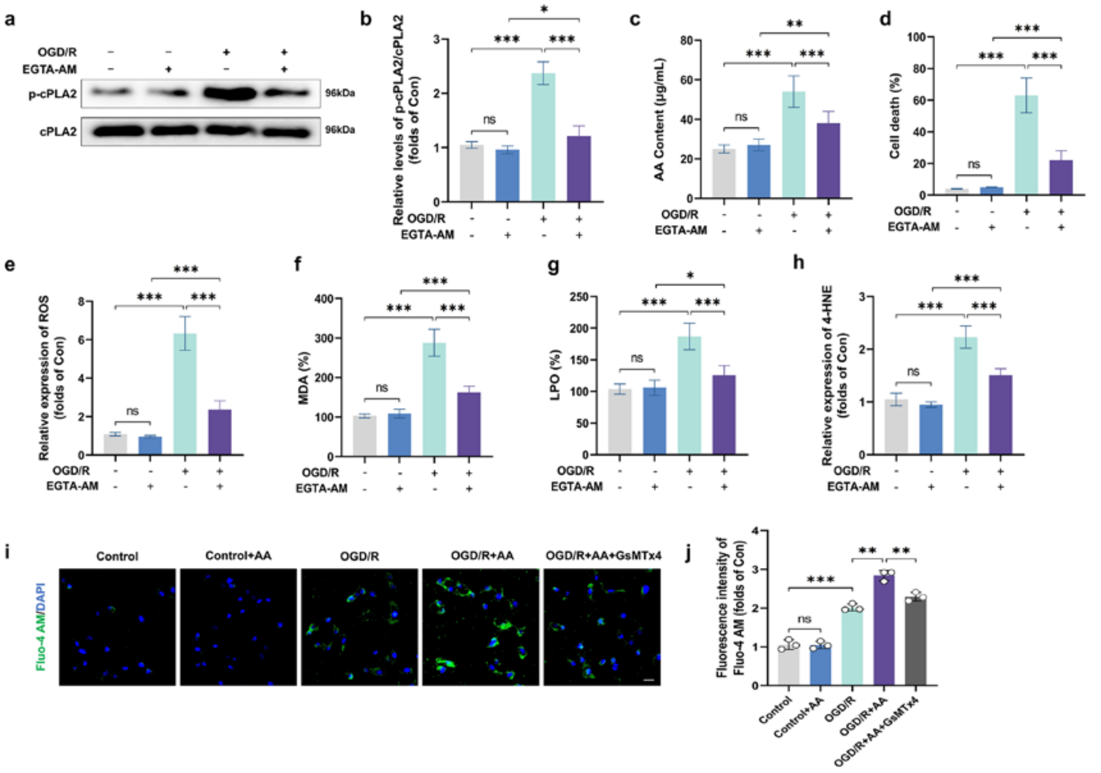

Supplementary Figure 5. The Impact of Piezo1 Activation on Ferroptosis in MECs Through the Ca^{2+}-cPLA2-AA Pathway. (a) Immunoblots for p-cPLA2 and total cPLA2 protein expression. (b) Quantitative assessment of p-cPLA2 to cPLA2 levels. (c) Measurement of AA content. (d) Further quantification of cell death. (e) ROS content displayed as a histogram. (f-h) Histograms showing the contents of 4-HNE, MDA and LPO. (i) Immunofluorescence images showing staining for Fluo-4 AM, scale bars at 100 μm. (j) The average optical density of Fluo-4 AM is presented. Data overview: presented as means ± SD. Significance notation: "ns" indicates no significant difference; Statistical significance levels: *(P < 0.05), **(P < 0.01) and ***(P < 0.001).

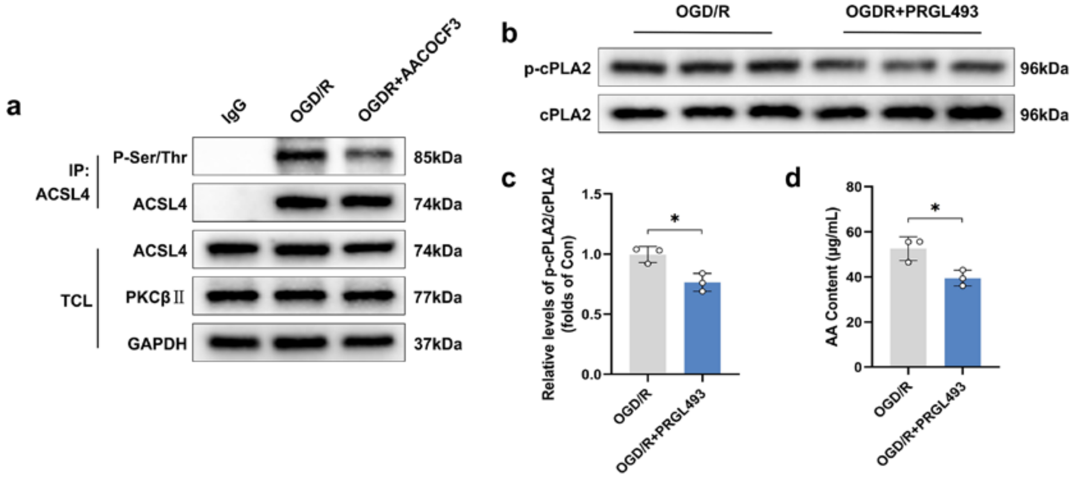

Supplementary Figure 6. Feedback regulation between ACSL4 and cPLA2. (a) Western blots showing phosphorylation of Ser/Thr residues and ACSL4 protein expression. (b) Immunoblots for p-cPLA2 and total cPLA2 protein expression. (c) Quantitative assessment of p-cPLA2 relative to total cPLA2 protein. (d) Measurement of AA content. Data overview: presented as means ± SD. Statistical significance levels: *($P < 0.05$).

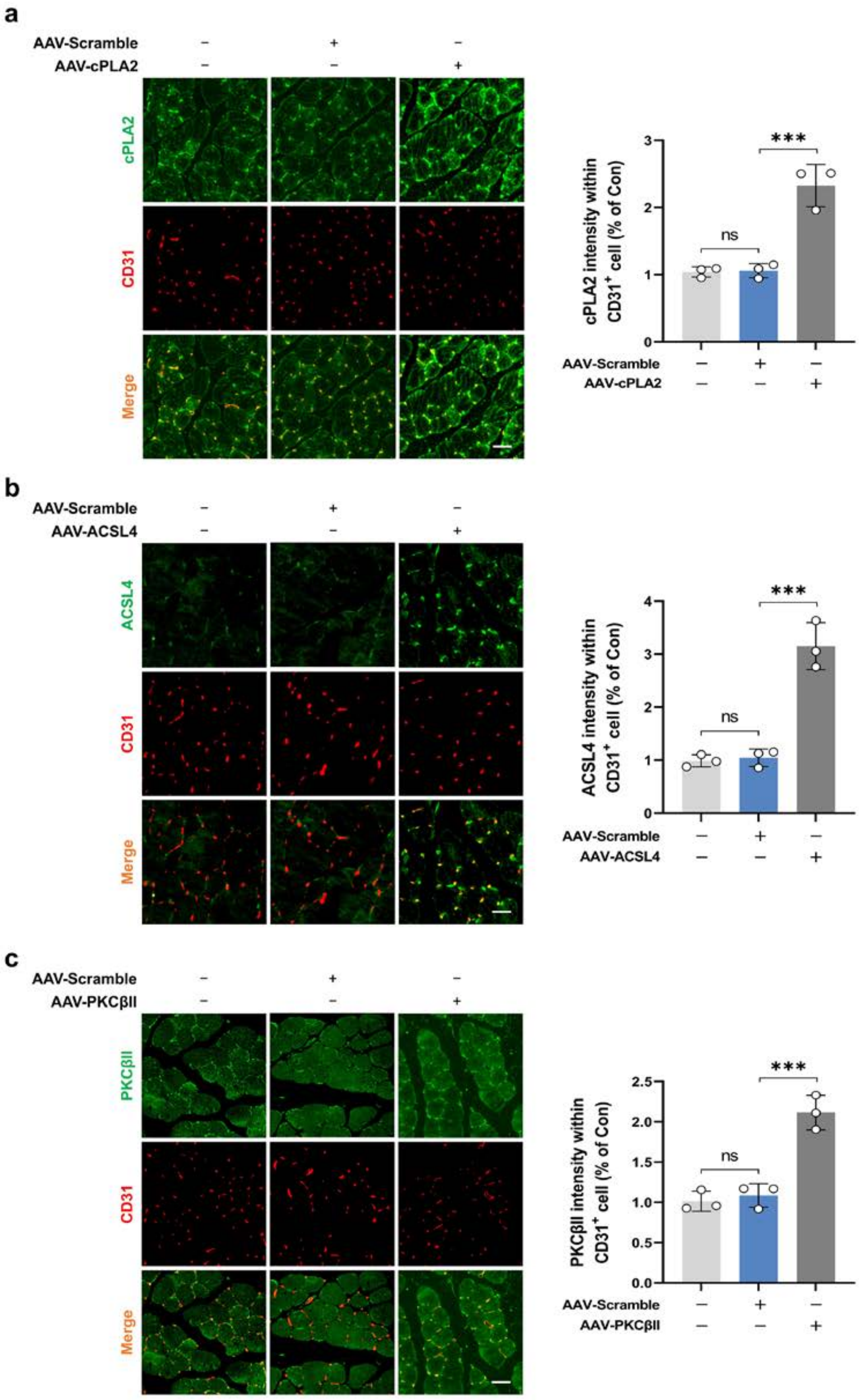

Supplementary Figure 7. (a) Representative immunofluorescence images of mouse tissue sections stained for cPLA2 (green) and the endothelial marker CD31 (red), with merged images shown in the bottom panels. Tissues were collected from mice

injected with AAV-Scramble or AAV-cPLA2 as indicated. And the quantification of cPLA2 fluorescence intensity within $CD31^+$ endothelial cells. (b) Representative immunofluorescence images of mouse tissue sections stained for ACSL4 (green) and the endothelial marker CD31 (red), with merged images shown in the bottom panels. Tissues were collected from mice injected with AAV-Scramble or AAV-ACSL4 as indicated. And the quantification of ACSL4 fluorescence intensity within $CD31^+$ endothelial cells. (c) Representative immunofluorescence images of mouse tissue sections stained for PKCβII (green) and the endothelial marker CD31 (red), with merged images shown in the bottom panels. Tissues were collected from mice injected with AAV-Scramble or AAV-PKCβII as indicated. And the quantification of PKCβII fluorescence intensity within $CD31^+$ endothelial cells. Data overview: presented as means ± SD. Significance notation: "ns" indicates no significant difference; Statistical significance levels: ***($P < 0.001$).
